# Infiltrating macrophages amplify doxorubicin-induced cardiac damage: role of catecholamines

**DOI:** 10.1007/s00018-023-04922-5

**Published:** 2023-10-11

**Authors:** Jessica Gambardella, Gaetano Santulli, Antonella Fiordelisi, Federica Andrea Cerasuolo, Xujun Wang, Nella Prevete, Eduardo Sommella, Roberta Avvisato, Antonietta Buonaiuto, Giovanna Giuseppina Altobelli, Laura Rinaldi, Francesco Chiuso, Antonio Feliciello, Fabrizio Dal Piaz, Pietro Campiglia, Michele Ciccarelli, Carmine Morisco, Junichi Sadoshima, Guido Iaccarino, Daniela Sorriento

**Affiliations:** 1 Department of Advanced Biomedical Sciences, Federico II University, Naples, Italy; 2 Department of Medicine (Cardiology) and Department of Molecular Pharmacology, Wilf Family Cardiovascular Research Institute, Albert Einstein College of Medicine, Montefiore University Hospital, New York, USA; 3 Hypertension Research Center (CIRIAPA), Federico II University, Naples, Italy; 4 Department of Translational Medical Sciences, Federico II University, Naples, Italy; 5 Institute of Experimental Endocrinology and Oncology (IEOS), CNR, Naples, Italy; 6 Department of Pharmacy, University of Salerno, Fisciano (Salerno), Italy; 7 Department of Molecular Medicine and Medical Biotechnology, Federico II University, Naples, Italy; 8 Department of Medicine, Surgery and Dentistry “Scuola Medica Salernitana”, University of Salerno (Salerno), Baronissi, Italy; 9 Department of Cell Biology and Molecular Medicine, New Jersey Medical School, Rutgers University, Newark, New Jersey, USA

**Keywords:** Doxorubicin, Macrophages, p53, Mitochondria, Mitophagy, β-AR, Catecholamines

## Abstract

**Background:**

The functional contribution of non-myocyte cardiac cells, such as inflammatory cells, in the setup of heart failure in response to doxorubicin (Dox) is recently becoming of growing interest.

**Objectives:**

The study aims to evaluate the role of macrophages in cardiac damage elicited by Dox treatment.

**Methods:**

C57BL/6 mice were treated with one intraperitoneal injection of Dox (20 mg/kg) and followed up for 5 days by cardiac ultrasounds (CUS), histological, and flow cytometry evaluations. We also tested the impact of Dox in macrophage-depleted mice. Rat cardiomyoblasts were directly treated with Dox (D-Dox) or with a conditioned medium from cultured murine macrophages treated with Dox (M-Dox).

**Results:**

In response to Dox, macrophage infiltration preceded cardiac damage. Macrophage depletion prevents Dox-induced damage, suggesting a key role of these cells in promoting cardiotoxicity. To evaluate the crosstalk between macrophages and cardiac cells in response to DOX, we compared the effects of D-Dox and M-Dox in vitro. Cell vitality was lower in cardiomyoblasts and apoptosis was higher in response to M-Dox compared with D-Dox. These events were linked to p53-induced mitochondria morphology, function, and autophagy alterations. We identify a mechanistic role of catecholamines released by Dox-activated macrophages that lead to mitochondrial apoptosis of cardiac cells through β-AR stimulation.

**Conclusions:**

Our data indicate that crosstalk between macrophages and cardiac cells participates in cardiac damage in response to Dox.

**Graphical abstract:**

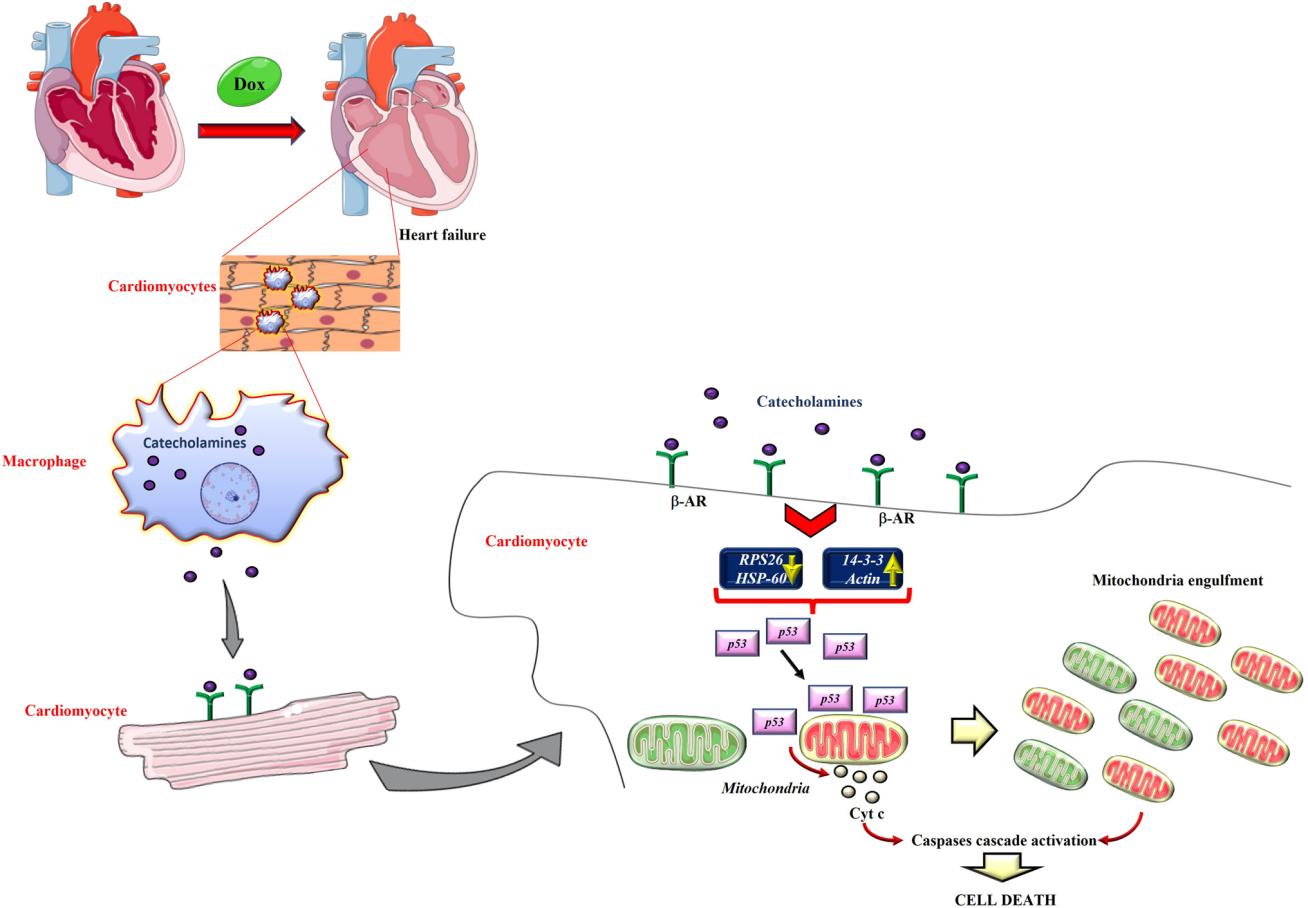

**Supplementary Information:**

The online version contains supplementary material available at 10.1007/s00018-023-04922-5.

## Introduction

Doxorubicin (Dox) is among the most effective anticancer agents but cumulative doses cause cardiotoxicity and irreversible heart failure (HF). Direct toxicity of Dox on cardiomyocytes is considered the most relevant mechanism of failure in anthracycline-treated patients due to the impairment of several intracellular pathways [1]. However, growing interest focuses on non-myocyte cardiac cells that hold the potential to modulate and amplify toxicity in cardiac myocytes [2, 3]. Recently, it has been suggested that macrophages could infiltrate the heart in response to chronic treatment with Dox [4]. Resident/infiltrating macrophages are known to regulate cardiac remodeling in response to injury through crosstalk with cardiac cells [5, 6] and participate in the development and progression of Dox-induced HF by regulating the release of inflammatory cytokines [7]. Transgenic mice bearing the deletion of the toll-like receptor (TLR-2) gene show a preserved cardiac function and a higher survival rate in response to Dox [8]. Also, oxidative stress, inflammation, and apoptosis inhibitors, like enoxaparin, are cardioprotective in response to Dox [9]. Overall, these findings point to macrophages as a potential contributor to Dox-dependent cardiac damage. Catecholamines represent an important regulator of cardiac function and structure [10], and, interestingly, macrophages possess the enzymatic machinery to synthesize and release catecholamines [11]. Therefore, the role of these neurotransmitters in the crosstalk between macrophages and cardiac cells in Dox-induced HF is reasonable. Based on such background, we explored whether crosstalk between macrophages and cardiac cells could affect Dox-induced damage in an acute model of cardiotoxicity.

## Materials and methods

A detailed description of methodologies is available in Supplemental Appendix.

## Results

### Macrophages infiltrate the heart in response to Dox

**Fig. 1:**
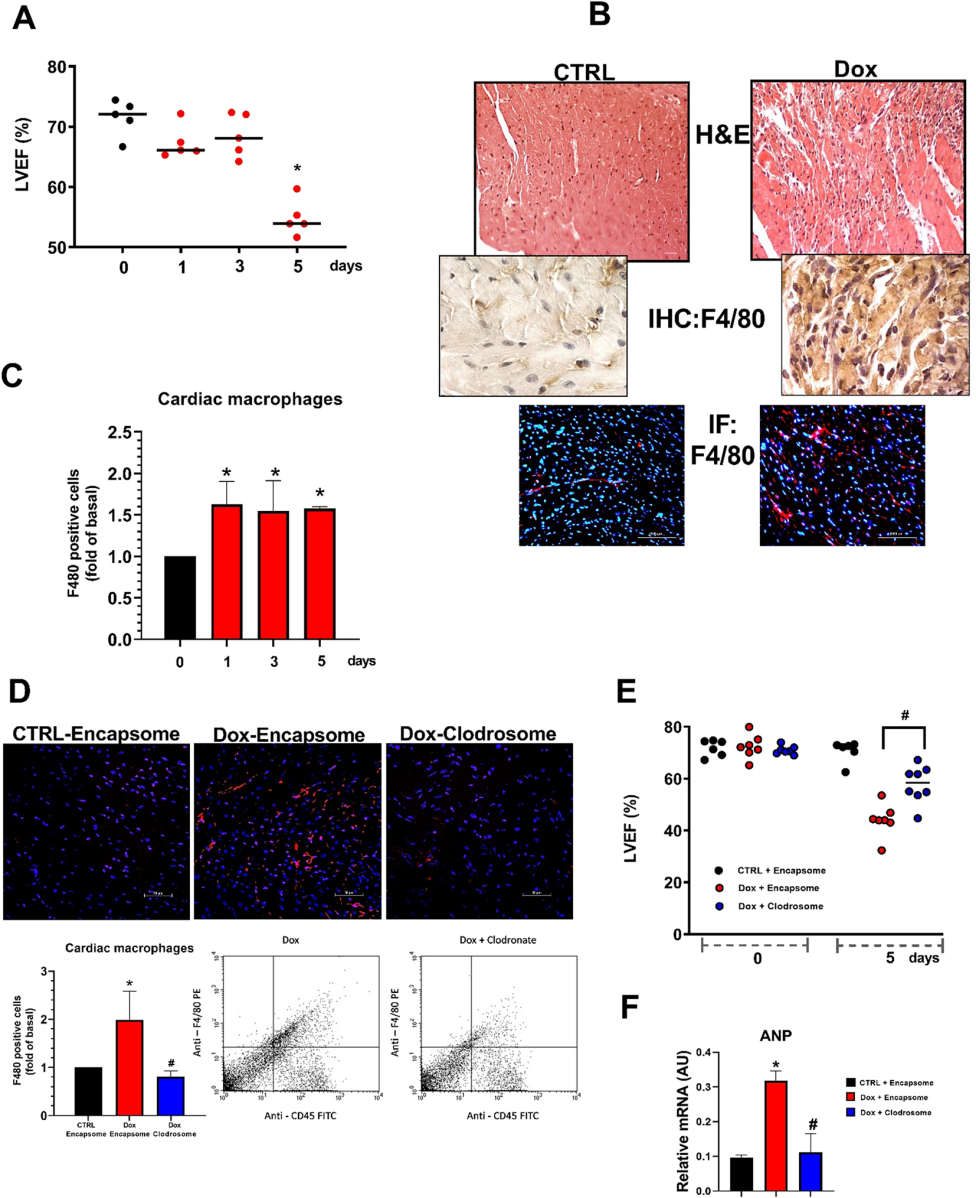
Macrophage infiltration in the heart in response to Dox. **a** Cardiac function was evaluated by echocardiography at different time points. The ejection fraction was reduced 5 days after treatment. Results are shown as mean ± SEM; *p < 0.05 vs “time zero” (*n* = 5). **b** Paraffin-embedded sections of heart were stained with hematoxylin and eosin (magnification × 200) or processed for immunohistochemistry (magnification × 600) and immunofluorescence (magnification × 400). Representative images of F4/80 staining reveal macrophage infiltration. **c** Hearts were processed for flow cytometry to evaluate F4/80 positive cells. The results are shown as mean ± SEM; *p < 0.05 vs “time zero” (*n* = 4). **d** Mice were pre-treated with Clodrosome or Encapsome and then treated with Dox for 5 days. Macrophages depletion was confirmed by both immunofluorescence (magnification × 400) and flow cytometry analysis, *p < 0.05 vs “CTRL + Encapsome”; ^#^*p* < 0.05 vs “Dox + Encapsome” (*n* = 4). **e** Cardiac function was evaluated in Clodrosome and Encapsome treated mice in response to Dox. Clodrosome ameliorated cardiac function. ^#^*p* < 0.05 vs “Dox + Encapsome”. **f** Dox-dependent increase in ANP gene expression was significantly reduced by Clodrosome. *p < 0.05 vs “CTRL + Encapsome”; ^#^*p* < 0.05 vs “Dox + Encapsome”

Mice were treated with an intraperitoneal injection of Dox and followed up to evaluate cardiac function; hearts were collected at different timepoints for biochemical and histological evaluations (Fig. S1A). A significant reduction in systolic function was detected on day 5 (Fig. 1a) which was mirrored by the increase in atrial natriureti peptide (ANP) gene expression (Fig. S1B). To evaluate the inflammatory phenotype in response to Dox, histological analysis was performed. Hematoxylin–eosin (H&E) and F4/80 staining revealed the presence of cellular infiltrate which mainly included macrophages (Fig. 1b). This finding is in line with results in a chronic model of Dox-dependent cardiotoxicity (Fig. S1C and [4]). The timing of heart inflammation in response to Dox was evaluated by flow cytometry at different time points. A significant increase in cardiac macrophages was detected since day 1 (Fig. 1c), with a net polarization toward M1 subtype (Fig. S1D), suggesting that Dox-induced macrophage infiltration in the heart is an event that precedes cardiac dysfunction. To confirm the role of macrophages in the development of cardiotoxicity, we depleted macrophages by using clodronate liposomes. Both flow cytometry and histological analysis showed that liposome treatment induced over 50% reduction of macrophages in the heart (Fig. 1d) and this was associated with an amelioration of cardiac function in Clodrosome treated mice as demonstrated by the increase in EF (Fig. 1e) and reduction in ANP gene expression (Fig. 1f). These data further support that macrophages are a key determinant in the development of cardiac dysfunction in response to Dox.

### Comparing direct and indirect effects of Dox in cardiac cells

**Fig. 2:**
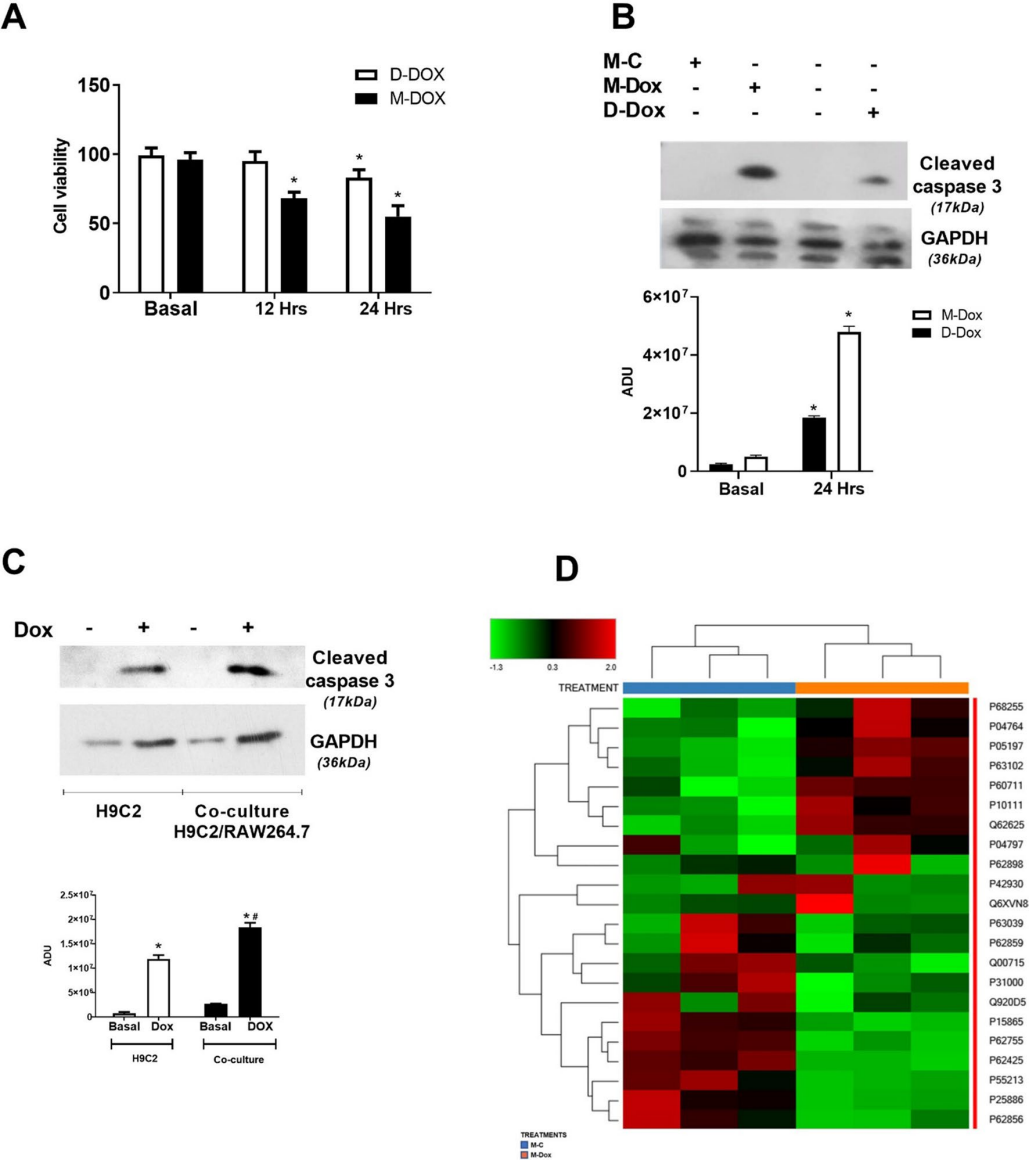
Effects of M-Dox and D-Dox treatments. **a** Cell viability was evaluated at 12 and 24 h. M-Dox reduced cell viability at 12 and 24 h while D-Dox induced a late reduction at 24 h. The results are shown as mean ± SEM in the bar graph; *p < 0.05 vs Basal. **b** Apoptosis was evaluated by western blot. Cleaved caspase 3 levels increased in both experimental conditions with a higher increase in M-Dox treated cells. Densitometric analysis is shown in the bar graph; *p < 0.05 vs Basal. **c** We performed co-culture experiments with RAW264.7 and H9C2 using Transwell permeable support. RAW264.7 were plated on the upper compartment of the Transwells and treatment was performed with Doxorubicin (10 μg/ml). Protein content of H9C2 was analyzed by western blot; *p < 0.05 vs Basal; ^#^*p* < 0.05 vs H9C2. **d** Mass spectrometry analysis was performed in H9C2 whole lysates, proteins that are up- or down-regulated in response to M-Dox are reported in a heat map

To evaluate macrophage-dependent cardiac cell damage, we treated H9C2 cardiomyoblasts directly with Dox (referred to as *D-Dox*) or with a conditioned medium derived from Dox-activated macrophages (referred to as *M-Dox*) (Fig. S2A). Macrophages were sensitive to Dox treatment in a time dependent manner (Fig. S2B). Both D-Dox and M-Dox treatments reduced cell viability (Fig. 2a) and increased cleaved caspase 3 levels at 24 h (Fig. 2b), but these effects were higher and earlier (12 h) in M-Dox treated cells. These findings strongly suggest the existence of a facilitation mechanism of death that is induced by macrophages. Accordingly, the co-culture of RAW264.7 and H9C2 further increased cell death of H9C2 compared with direct treatment of these cells with Dox (Fig. 2c). To identify this mechanism, we evaluated the proteomic profile of M-Dox treated H9C2. Several proteins involved in metabolic processes exhibited differential expression in cell lysates from M-Dox and M-C groups (Supplementary Table 1), and most of them are part of the interactome of p53 (Figs. 2d, S3). 14-3-3, Actin-1, RPS26, and HSP-60 are known regulators of p53 stability and activity [12]. In our experimental conditions, their alterations (increase in 14-3-3 and Actin-1; reduction in RPS26 and HSP-60) suggest the involvement of p53 in M-Dox-dependent damage.

### The role of p53 in M-Dox-dependent damage

**Fig. 3:**
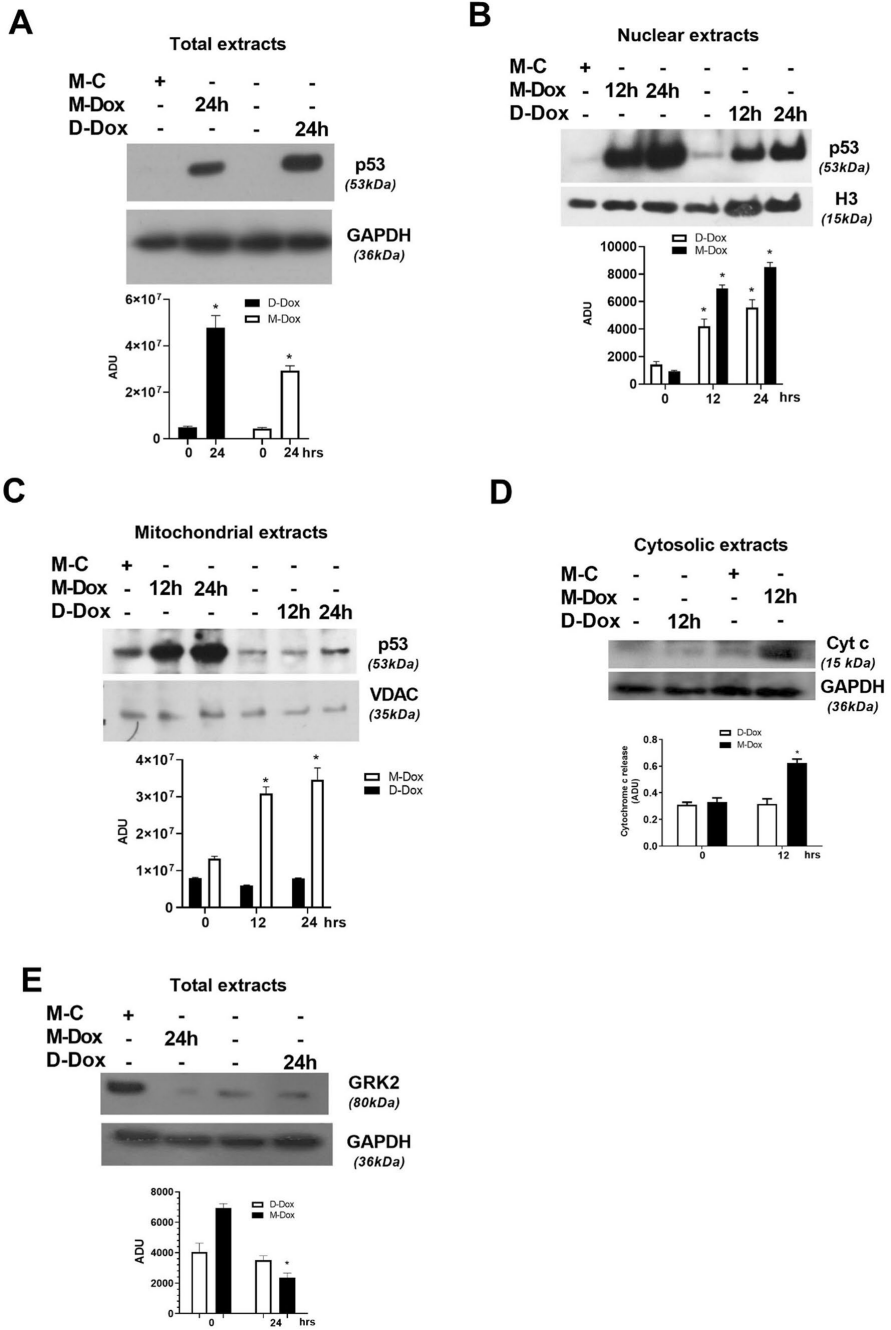
Effects of M-Dox and D-Dox on p53. **a** Mitochondrial levels of p53 were detected by western blot. They were significantly and early increased in response to M-Dox. Densitometric analysis is shown in the bar graph; *p < 0.05 vs “time zero”. **b** Cytochrome c levels were evaluated in the cytosolic extract by western blot. An early increase in Cytochrome c was found early in M-Dox treated cells. Densitometric analysis is shown in the bar graph; *p < 0.05 vs “time zero”. The levels of p53 were evaluated by western blot in whole lysates (**c**) and nuclear extracts (**d**). In both experimental conditions, whole and nuclear p53 levels were increased. Densitometric analysis is shown in the bar graph; *p < 0.05 vs “time zero”. **e** GRK2 levels were evaluated by western blot in cells treated with M-Dox and D-Dox for 24 h. The kinase levels were reduced in response to M-Dox but not D-Dox. Densitometric analysis is shown in the bar graph; *p < 0.05 vs “time zero”

We then examined p53 levels in the subcellular compartments of H9C2 treated with M-Dox or D-Dox. Both D-Dox and M-Dox treated cells increased total (Fig. 3a) and nuclear (Fig. 3b) levels of p53, suggesting the involvement of the transcriptional activity of p53 in cardiac cell damage in both experimental conditions. Interestingly, M-Dox, but not D-Dox, induced an accumulation of p53 in mitochondria (Fig. 3c), accompanied by the early release of cytochrome c (Fig. 3d) in the cytosol. As in other forms of HF, mounting evidence has pointed to adrenergic dysregulation in Dox-induced cardiotoxicity [13, 14]. To explore the adrenergic signaling in our model and to further strengthen the evidence that M-Dox causes a mitochondrial mechanism of death in H9C2, we measured the levels of GRK2, a known regulator of βAR signaling which can also modulate both p53 [15] and mitochondrial apoptosis [16–19]. We found that M-Dox triggered a significant downregulation of GRK2 levels (Fig. 3e), in line with mitochondrial damage and the increase in p53 levels. Strikingly, an opposite effect was revealed in response to D-Dox, thus corroborating the activation of a different mechanism of apoptosis that does not involve mitochondrial p53. Altogether these data imply the involvement of macrophages in Dox-dependent cardiotoxicity and point to p53-dependent mitochondrial alterations as a potential mechanism of macrophage-mediated amplification of H9C2 cell death.

### M-Dox regulates mitochondrial morphology and functions

**Fig. 4:**
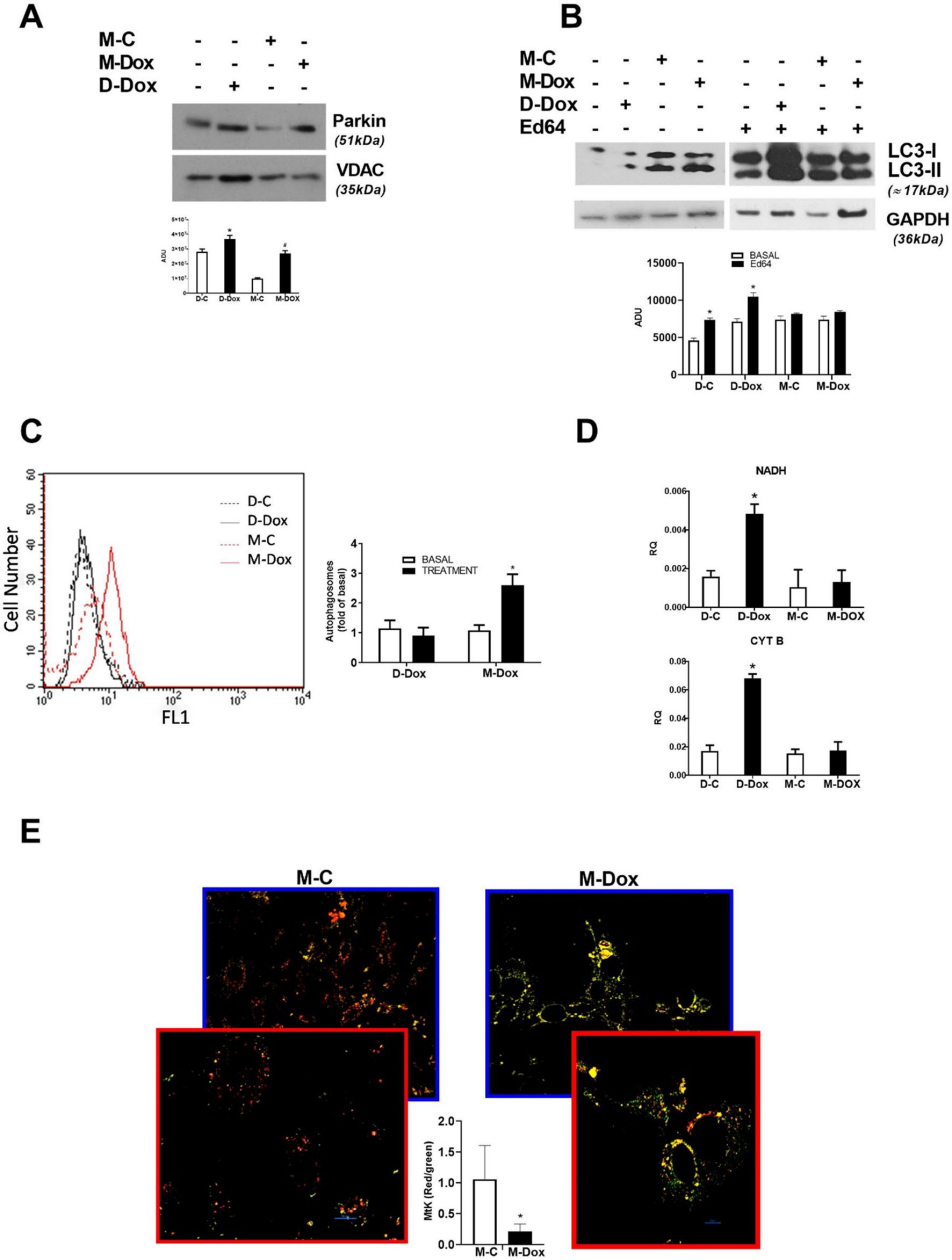
Alterations of mitophagy in M-Dox treated cells. **a** Mitochondrial levels of Parkin were evaluated by western blot. Densitometric analysis is shown in the bar graph; *p < 0.05 vs D-C; ^#^*p* < 0.05 vs M-C. **b** Levels of LC3-II were evaluated by western blot. LC3-II levels were unchanged in response to M-Dox both in the absence and presence of E46d. Densitometric analysis is shown in the bar graph; *p < 0.05 vs M-C. **c** Autophagosomes were quantified at a flow cytometer. The graph shows autophagosomes in response to D-C (dotted black line), D-Dox (continuous black line), M-C (dotted red line), and M-Dox (continuous red line). **d** Mitochondrial biogenesis was evaluated by real-time PCR. M-Dox blocked mitochondrial biogenesis. Data are shown as mean ± SEM in the bar graph. **e** Live images of M-C and M-Dox treated H9C2 expressing AAV-Mito-Keima. Scale bar, 10 μm. The graph shows the ratio of red vs green-labeled cells. *p < 0.05 vs M-C

Since mitochondria are critical targets of Dox, we evaluated the effects of M-Dox on mitochondrial function. To explore the effects of M-Dox on mitophagy, we evaluated mitochondrial Parkin levels, LC3-II levels and autophagosome accumulation. Parkin translocated to mitochondria in both conditions but the levels were higher in response to M-Dox suggesting a higher number of damaged mitochondria or the lack of their elimination (Fig. 4a). LC3-II levels were increased in D-Dox treated cells both in the presence and absence of Ed64, an inhibitor of autophagy, but were not modified in response to M-Dox (Fig. 4b). This finding was further supported by the accumulation of autophagosomes (Fig. 4c) and the resulting block of mitochondrial biogenesis in response to M-Dox (Fig. 4d). These data suggest a failure in the autophagic flux only in response to M-Dox. A further confirm derived from the use of an adeno-associated virus (AAV) harboring mitochondria-targeted mKeima (AAV-Mito-Keima). M-Dox reduced the ratio of red (acidic) to green (neutral) fluorescence, indicating that most mitochondria were not directed to lysosomal degradation (Fig. 4e). To verify whether defects in autophagy could be responsible for M-Dox-induced cell damage, we evaluated the effects of Urolithin-A, an inducer of autophagy. Urolithin-A significantly increased LC3-II levels in response to M-Dox (Fig. S4A) and reduced cleaved caspase 3 levels (Fig. S4B). These data confirm the involvement of autophagy alterations in M-Dox-dependent cell damage and suggest autophagy inducers as potential therapeutic strategy. Mitochondrial transplantation is used as a therapeutic approach for cardioprotection [20]. Given the accumulation of damaged mitochondria, we tested whether transplantation of healthy mitochondria in M-Dox treated cells could be effective to interrupt apoptosis. Opposite, mitochondrial transplantation worsened apoptosis (Fig. S4C) suggesting that M-Dox-dependent mitochondrial apoptosis is activated through a signaling process rather than a primary mitochondrial failure.

**Fig. 5:**
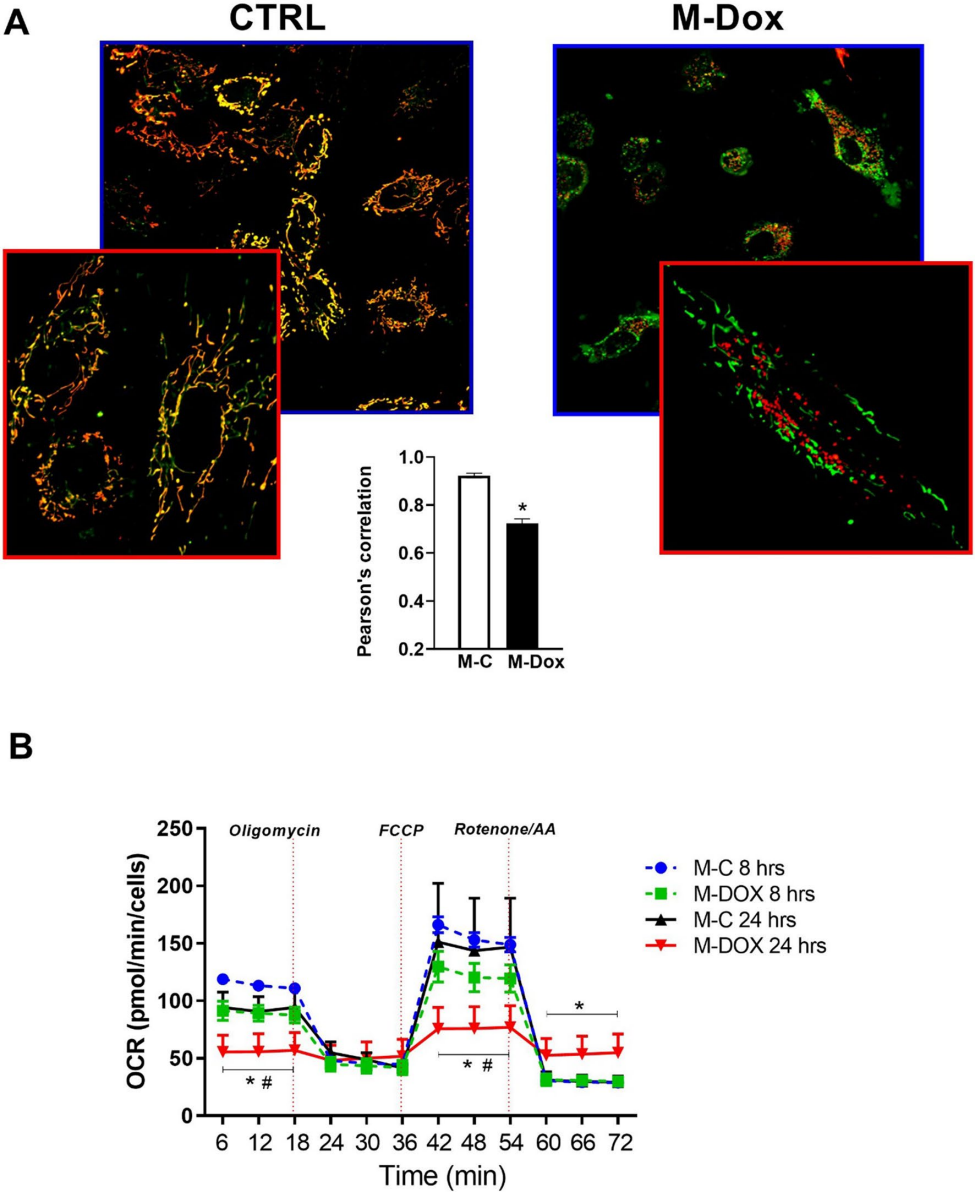
Mitochondria alterations in response to M-Dox. **a** Cells treated with M-Dox were analyzed at confocal microscopy after staining with mitotracker and TMRE. Most M-Dox treated cells showed an impaired mitochondrial morphology. The graph shows the Pearson’s correlation that indicates a net reduction in TMRE staining in M-Dox samples. *p < 0.05 vs M-C. **b** Mitochondrial oxygen consumption of M-Dox treated cells was analyzed at 8 and 24 h by Seahorse, resulting significantly reduced; ^#^*p* < 0.05 vs M-C 8 h, *p < 0.05 vs M-C 24 h

To evaluate mitochondrial morphology and function, we used Mitotracker to label all mitochondria and TMRE to discriminate the efficient ones. In M-C treated cells, mitochondria are organized in a fused network and the two mitochondrial indicators overlap (yellow) (Fig. 5a). On the other hand, M-Dox treated cells displayed altered mitochondrial morphology with a net prevalence of fixed organelles compared with D-Dox (Fig. 5a and Fig. S5). In M-Dox cells, mitochondria failed to sequester TMRE (green) or were hyperpolarized (red) indicating, in both cases, alterations of mitochondrial membrane potential, in line with the release of cytochrome c from these organelles (Fig. 5a). These morphological and functional alterations were associated with reduced mitochondrial oxygen consumption at both 8 and 24 h (Fig. 5b).

### Dox-activated macrophages produce catecholamines

**Fig. 6:**
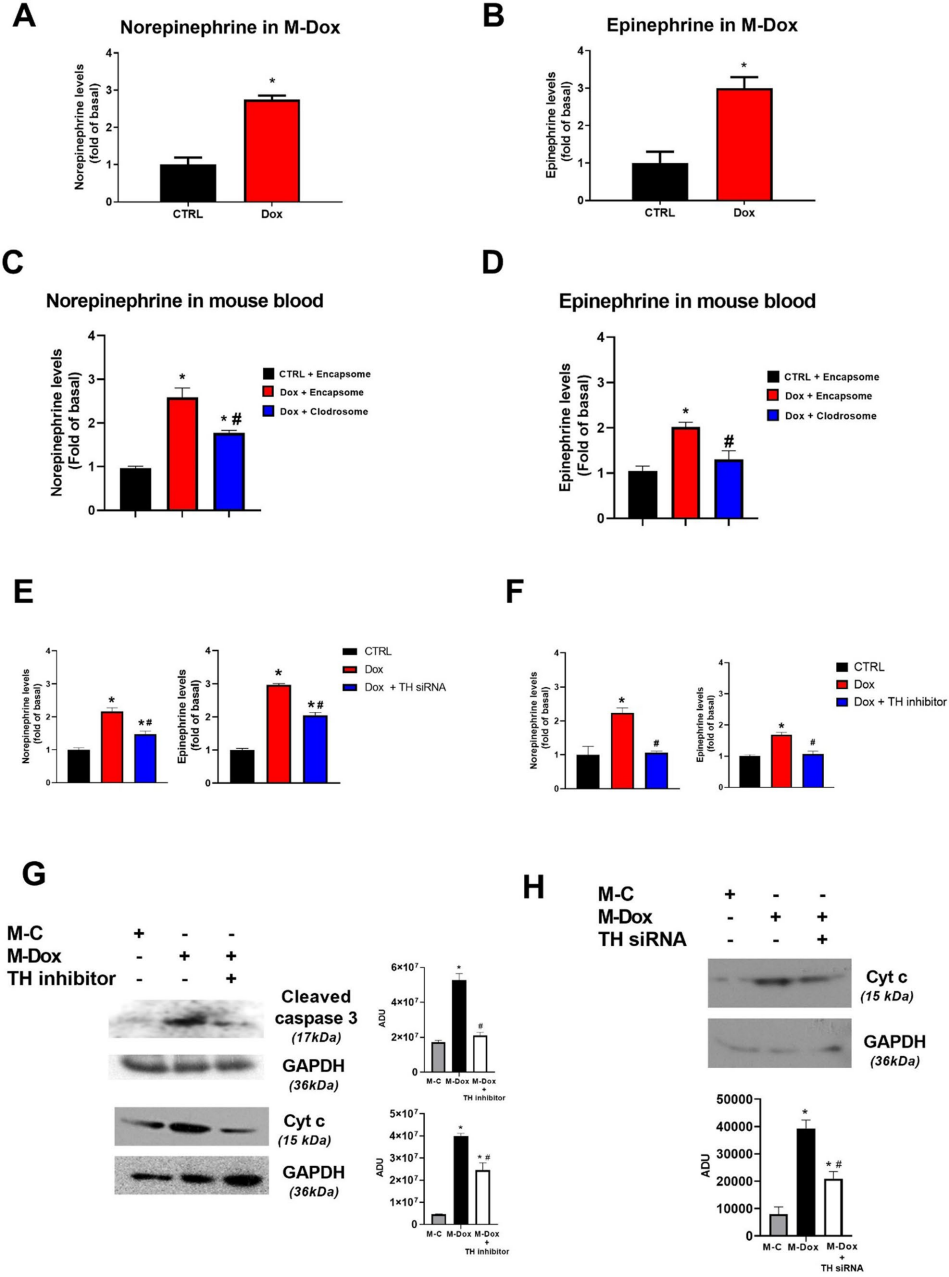
Dox-activated macrophages release catecholamines. Norepinephrine (**a**) and Epinephrine (**b**) levels were evaluated in the culture medium of macrophages treated with Dox for 24 h (**a**, **b**) by ELISA assay. * p < 0.05 vs CTRL. **c**, **d** Norepinephrine (GC) and Epinephrine (HD) levels were evaluated by ELISA assay in plasma from Clodrosome and Encapsome treated mice after 5 days of Dox treatment. *p < 0.05 vs “CTRL + Encapsome”; ^#^*p* < 0.05 vs “Dox + Encapsome”. **e**–**h** The inhibition of TH enzyme activity (**e**) or its silencing (**f**) reduced catecholamines release in the culture medium. The inhibition of TH enzyme activity is associated with reduced cleaved caspase 3 and cytochrome c levels (**g**). TH gene deletion confirmed the reduction in cytochrome release from mitochondria (**h**) supporting the suggested mechanism. Densitometric analysis is shown in the bar graph; *p < 0.05 vs M-C; ^#^*p* < 0.05 vs M-Dox

Since Dox was removed from M-Dox (Fig. S2C), all these mitochondrial alterations cannot depend on Dox, indeed M-Dox and D-Dox exerted different effects. In vitro in M-Dox medium, no significant differences were detected in cytokine production at 24 h (data not shown), suggesting that cytokines are slightly involved in “triggering” cardiac cell death in vitro even if we do not exclude their participation in the progression of cell damage. Thus, we focused on the ability of macrophages to produce catecholamines [21] which are known to amplify cardiac damage both in the conditioned medium from Dox-activated macrophages and in blood samples from Dox-treated mice. Catecholamines were increased in medium from Dox-treated RAW264.7 (Fig. 6a, b) as well as in blood from mice treated with Dox (Fig. 6c, d). Macrophage depletion in vivo significantly reduced the levels of circulating catecholamines (Fig. 6c, d) supporting the crucial role of catecholamines in M-Dox and its relevance in vivo in cardiotoxicity. Accordingly, the block of catecholamine synthesis in macrophages through the inhibition of TH enzyme activity (Fig. 6e) or the silencing of TH mRNA (Fig. 6f and Fig. S6) was able to prevent cell death and cytochrome c release induced by M-Dox (Fig. 6g, h) supporting the key role of catecholamines produced by Dox-activated macrophages in cardiac cell damage. To further confirm these data, we also evaluated the effects of direct epinephrine treatment or beta-blocking in H9C2. The addition of epinephrine in the culture medium of H9C2 increased mitochondrial translocation of p53 (Fig. S7A) in a dose-dependent manner suggesting its involvement as a trigger of macrophage-dependent p53 activation. Since catecholamines activate β-adrenergic receptor (β-AR) signaling, we inhibited β-ARs by metoprolol and evaluated apoptosis. M-Dox increased cleaved caspase 3 levels but the inhibition of β-ARs reverted this effect (Fig. S7B) confirming the deleterious effect of catecholamines via β-ARs signaling. The activation of adrenergic signaling in response to M-Dox was further proved by the accumulation of GRK2 on the plasma membrane (Fig. S7C) and its reduction in mitochondria (Fig. S7D). To confirm the physio-pathological relevance of our findings, we tested the main findings of this study in cardiomyocytes and hearts isolated from mice (Fig. S8) and evaluated catecholamines production also in peritoneal macrophages in response to Dox (Fig. S9).

## Discussion

In this study, we demonstrate that macrophages infiltrating the heart actively participate in Dox-dependent cardiac damage through the release of catecholamines and stimulation of βARs, which in turn activate p53 and lead to mitochondrial dysfunction and cardiomyoblast apoptosis. Our results are well in agreement and further exploit the previous literature regarding Dox-induced activation of resident macrophages and infiltration of circulating monocytes in a chronic model of cardiotoxicity [4]. Based on these findings, here we used an acute model of cardiotoxicity, a high reproducible model of cardiac damage [22, 23], and focused on the early response to Dox providing experimental proof of the detrimental effects of infiltrating macrophages through the release of catecholamines. While the infiltration of proinflammatory macrophages in the heart in response to Dox has been suggested, the pathophysiological mechanism was not investigated [4, 24]. In our study, we prove for the first time, also through depletion, that infiltrating macrophages are key determinants in the development of cardiotoxicity. Here, we analyzed functional crosstalk between macrophages and cardiomyoblasts, a validated cellular model to explore in vitro the functional crosstalk of cardiac cells contributing to cardiac homeostasis and function [25] and evaluate energy metabolism patterns [26]. However, key experiments were also confirmed in cardiomyocytes and hearts, as well as in peritoneal macrophages, to underline the physiological relevance of our results. Several studies point to the involvement of p53 in Dox-dependent cardiac damage: the genetic deletion of the p53 gene [27] or its pharmacological inhibition [28] are cardioprotective in response to Dox, as well as its mutation with dominant-interfering activities [29]. Generally, p53 exerts its effects in a transcription-dependent manner in the nucleus. Indeed, in cultured neonatal rat cardiomyocytes, Dox increases the nuclear translocation of p53 and induces cell death [30]. However, growing evidence also suggests the involvement of p53 in mitochondrial mechanisms of death [31, 32]. In cardiomyoblasts, both D-Dox and M-Dox induce cell damage and death through nuclear p53 activity suggesting the involvement of the transcriptional activity of this protein in cardiotoxicity. However, M-Dox also activates p53-dependent mitochondrial mechanisms of apoptosis. Accordingly, M-Dox, but not D-Dox, increases cytochrome c release causing alterations in mitochondrial morphology and function. As extensively described in the literature, direct Dox treatment induces mitochondrial dysfunction in cardiomyocytes thus we did not explore this issue. Here, we found that macrophages further damage cardiac cells through the release of catecholamines which induces p53-dependent mitochondrial apoptosis. Further confirmation comes from the downregulation of GRK2 in response to M-Dox. Several studies demonstrated the protective effects of this kinase in mitochondria by promoting mitochondrial biogenesis and increasing ATP production in different cellular subtypes [16, 18, 19]. Moreover, GRK2 counter-regulates p53 expression in cancer cells [15]. M-Dox reduces the total and mitochondrial levels of GRK2, removing its protective effect in mitochondria and increasing p53 levels. GRK2 levels are not modified by the direct treatment with Dox, suggesting that the drug itself does not affect the kinase. Thus, the amplification of H9C2 damage in response to M-Dox is mediated by a paracrine factor released by macrophages which coactivates an additional death pathway. The proteomic analysis identifies an interesting network of modulated proteins that are involved in the regulation of metabolic processes and some of them are strongly associated with p53 stability and activity (14-3-3, Actin, RPS26, and HSP60). The 14-3-3 family can affect p53 stability and transcriptional activity through direct and indirect interactions; Actin interacts with p53 and favors its nuclear transport [33, 34]. These proteins are all increased in M-Dox treated cells suggesting their involvement in favoring the nuclear effects of p53. On the other hand, the knockdown of the ribosomal protein RPS26 induces p53 stabilization and activation and the deletion of Hsp60 stabilizes p53 leading to mitochondrial dysfunction and apoptosis [34, 35]. Accordingly, the levels of RPS26 and HSP60 were reduced in M-Dox treated cells, thus favoring mitochondrial p53 stability and activity. Hence, M-Dox activates mitochondrial pathways of apoptosis that join to the nuclear effects of p53.

Altogether, our findings suggest that macrophages are key players in the development of cardiotoxicity contributing to cardiac cell damage and death through functional crosstalk with cardiac cells. Several findings demonstrate that myeloid cell lines, including macrophages [21] and endothelial cells [36], produce catecholamines in response to different stimuli. The increase in catecholamines is a feature in stress-induced HF [37]. They activate two classes of adrenergic receptors (ARs) in the myocardium, the α-ARs, which generally exert a protective role, and β-ARs, whose effects are involved in the pathophysiology of HF [10]. Here, we show that both epinephrine and norepinephrine are increased in M-Dox medium as well as in blood from mice, and macrophage depletion significantly reduces the levels of circulating catecholamines in mice. In vitro, β-blocking is effective to recover from damage induced by M-Dox. This observation is in line with the protective role of β-blockers in anthracycline-induced cardiotoxicity in the clinical scenario [38] and points to catecholamines as an essential trigger of macrophages-dependent amplification of Dox-induced cardiac cell damage.

Mitochondrial dysfunction is considered a primary factor underlying the pathogenesis of cardiotoxicity such that the preservation of mitochondrial health appears a promising strategy to prevent this pathologic condition. Autophagy is a key mechanism to remove damaged cells and organelles (mitophagy) and maintain cell homeostasis and mitochondrial quality; alterations in this process are associated with several conditions, including cardiovascular diseases. In particular, autophagy recently emerged as a key player in doxorubicin-dependent cardiotoxicity and several mechanisms have been revealed underpinning alterations of the autophagic flux in response to Dox [39]. Among them, p53 affects mitophagy by regulating mitochondrial translocation of Parkin and its downregulation reverses this process [40]. Our data also show alteration of mitophagy in M-Dox treated cells which occurs in the final steps of the process with a significant accumulation of autophagosomes including dysfunctional mitochondria that can be neither removed nor replaced with active ones. These events cause a deleterious autophagic engulfment within the cell that we try to revert in different ways. Increasing mitochondrial mass by transplanting healthy mitochondria in cells is a novel therapeutic strategy to restore impaired mitochondrial function in pre-clinical and clinical models [20, 41]. However, our model is characterized by an unfavorable mitochondrial and cellular environment where the transfer of healthy mitochondria leads to their fast impairment. On the contrary, Urolithin A, a natural microflora-derived metabolite that can accelerate mitophagy and improve muscle health in pre-clinical models of aging [42, 43], accelerates mitochondrial dynamics and favors unhealthy mitochondria disposal.

## Conclusions

We demonstrated for the first time the crosstalk between macrophages and cardiac cells in response to Dox. Dox induces early recruitment of macrophages in the heart which, in turn, release catecholamines and activate cardiac β-ARs. This induces p53-dependent damage in mitochondria that activates caspases cascade and cell death. Our findings specifically support early β-blocker intervention targeting the detrimental macrophages to prevent, rather than to treat, Dox-induced cardiac damage. The use of β-blockers is the standard therapy to treat cardiotoxicity in oncologic patients and our findings support the use of low doses of β-blockers to treat oncologic patients undergoing anthracycline therapy with high cardiovascular risks (hypertension, previous cardiovascular events) before the expression of clinical signs of cardiotoxicity to prevent catecholamines-dependent activation of cardiac cell death. Nevertheless, our findings propose novel therapeutics such as Urolithin-A, whose beneficial effect on mitochondrial and cellular health in humans [44] supports the proof of concept that a specific dietary supplementation could be helpful in the treatment of those pathologic conditions characterized by mitochondrial dysfunction, including cardiotoxicity.

### Supplementary Information

Below is the link to the electronic supplementary material.

Supplementary file1 (DOCX 2795 KB)

## Data Availability

All data generated during this study are included in this published article.
