# Supplementary material for: Infiltrating macrophages amplify doxorubicin-induced cardiac damage: role of catecholamines
Source: Cell Mol Life Sci. 2023 Oct 11;80(11):323. doi: 10.1007/s00018-023-04922-5 (PMC10567889; doi:10.1007/s00018-023-04922-5)
Supplement: Supplementary file 1 — Supplementary file1 (DOCX 2795 KB) [file 18_2023_4922_MOESM1_ESM.docx]

**SUPPLEMENTARY MATERIAL**

**Materials and Methods**

**In vivo study**

Experiments were carried out in 20-week-old C57BL/6 mice of both sexes, which were purchased by Charles River. Mice were housed in the Animal Facility of the Department of Translational Medical Sciences of Federico II University of Naples (Italy) and Health Monitoring was performed according to FELASA guidelines. All mice were housed in a 12-hour light-dark cycle under pathogen-free conditions and had access to a commercial mouse diet and water ad libitum. All *in vivo* experimental protocols were approved by the Italian Ministry of Health (Prot. n. 971/2016-PR). The mice were randomly divided into two groups and treated with one intraperitoneal injection of 20 mg/Kg of doxorubicin or saline. In some experiments, a chronic administration of Dox was performed through 3 injections/week of 2,5 mg/Kg of Dox for two weeks. At indicated time points, cardiac ultrasounds were conducted, and then the mice were euthanized by cervical dislocation. Heart and blood were collected: ANP gene expression, F4/80 staining at flow cytometer, and histological analysis were performed. In a subgroup of mice, we performed macrophage depletion by Clodronate liposomes (Encapsula Nanosciences, SKU# CLD-8901). Mice were treated with Clodrosome (Clodronate liposomes, 10µl/g) or Encapsome (Control liposomes, 10µl/g). In particular, mice received 4 liposomes and 1 Dox intraperitoneal injection as follows: Day 1=liposome, Day 4=liposome, Day 6=liposome, Day 7=Dox, Day 8=liposome, Day 12= echocardiography, and heart collection. Macrophage depletion was confirmed by immunofluorescence analysis and flow cytometry.

**Echocardiography**

Transthoracic echocardiography was performed with a small‐animal high‐resolution imaging system (VeVo770, VisualSonics, Inc, Toronto, Canada) equipped with a 30‐MHz transducer (Real‐Time Micro Visualization, RMV‐707B). The mice, anesthetized with isofluorane (4% induction, 2% maintenance), were placed in a shallow left lateral decubitus position, with strict thermoregulation (37±1°C) to optimize physiological conditions and reduce hemodynamic variability. Fur was removed from the chest by application of a cosmetic cream (Veet, Reckitt Benckiser, Milan, Italy) to gain a clear image. LV end‐diastolic and LV end‐systolic diameters were measured at the level of the papillary muscles from the parasternal short‐axis view. Intraventricular septal and LV posterior wall thickness were estimated at end-diastole. LV fractional shortening was calculated as follows: LVFS = [(LVEDD − LVESD)/ LVEDD] × 100, where LVFS indicates LV fractional shortening; LVEDD, LV end‐diastolic diameter; and LVESD, LV end‐systolic diameter. LV ejection fraction was calculated automatically by the echocardiography system. All measurements were averaged on 10 consecutive cardiac cycles per experiment and were analyzed by one experienced investigator [1]. Transthoracic echocardiography was performed at 0, 1, 3, and 5 days after Doxorubicin treatment.

**Peritoneal macrophage isolation**

Macrophages were isolated by intraperitoneal (IP) injection of ice-cold PBS. A gentle massage of the peritoneum was performed to dislodge any attached cells into the PBS solution, which was then collected by a 5 ml syringe. After centrifugation, cells were plated in 6 cm dishes, non-adherent cells were washed out, and doxorubicin was added to select wells to final concentrations of 10µg/ml. The characterization of attached cells was performed by flow cytometry.

**Cardiomyocytes isolation**

Adult mice ventricular myocytes (AMVMs) were isolated from wild-type mice by a standard digestion procedure using the Langerdhoff system, as previously described[2].

**Histological analysis**

Paraffin-embedded sections of the heart were stained with hematoxylin-eosin or processed for immunohistochemistry as previously described [3]. For immunofluorescence analysis of heart samples, cryostat sections were fixed in cold methanol, blocked in BSA 1%, and incubated with specific primary (F4/80, 1:50 SantaCruz) and secondary antibodies (anti-mouse 1:100, SantaCruz) for 1 hour at room temperature.

**ELISA assay**

The release of catecholamines in the culture medium or blood samples was analyzed by ELISA assay (Cloud-Clone Corp.), accordingly to the manufacturer’s instructions. Data are shown as fold of basal.

**Cell culture**

Murine macrophages (RAW264.7) and rat cardiomyoblasts (H9C2) were cultured in Dulbecco's minimal essential medium (DMEM) supplemented with 10% fetal bovine serum (FBS) at 37 °C in 95% air – 5%CO2. In some experiments, RAW264.7 and H9C2 were co-cultured in Transwell permeable support (Corning) and treated with Dox (10 μg/ml). The in vitro model included two different treatments of H9C2: 1) conditioned medium from Dox-activated macrophages (referred to as ***M-Dox***) using conditioned medium from untreated macrophages (referred to as ***M-C***) as control; 2) direct treatment with Dox (10 μg/ml) (referred to as ***D-Dox***) using untreated cells (referred to as ***D-C***) as control. To avoid the possibility that M-Dox effect could depend on Doxorubicin which is still present in the culture medium, we removed Doxorubicin from M-Dox using Amicon Ultra-4 Centrifugal Filters (Millipore), as previously described [4-8]. Dox precipitated and accumulated in the filter; the upper and lower solutions were collected and combined and the absence of Dox was confirmed by mass spectrometry analysis (9) In some experiments, H9C2 were pre-treated with Ed64 (10µg/ml), an inhibitor of autophagy, and Urolithin A (50µM), an inducer of autophagy.

**Mouse immune cells FACS analysis**

Mice hearts were dissociated in single-cell suspension by using GentleMACS (Miltenyi). For specific immune cells, population recognition anti-CD45 and anti-F4/80 were used in combination for the detection of macrophages. An Anti-troponin antibody was used to discriminate cardiomyocytes. For discrimination of M1 and M2 macrophages anti-CD80, anti-MHC-II, anti-CD206, and anti-CCR2 Cells were analyzed with a FACS Fortessa using Diva software (BD Biosciences) or a FACSCalibur using CellQuest software (BD Biosciences) [10]. Cells were labeled with Cyto-Id (1:500), a specific autophagosome marker, and the fluorescent signal was analyzed by flow cytometry (FACSCalibur, BD Biosciences).

**Mitochondrial biogenesis**

Cell DNA was isolated using commercially available reagents (DNAzol-Invitrogen). Real-time quantitative polymerase chain reaction (RT-PCR) was performed on DNA to amplify two mitochondrial (cytochrome b, NADHd), and one nuclear (18 s). All values obtained were normalized to the values obtained for endogenous control. The reaction was visualized by Sybr Green Analysis (Applied Biosystem) on the StepOne instrument (Applied Biosystem). The results are expressed as the relative integrated intensity.

**Western blot**

Total, mitochondrial, and cytosolic lysates were prepared as described previously [11, 12]. Western blot analyses were performed as previously described [13, 14]. [13, 14]. Lysates were electrophoresed by SDS/PAGE and transferred to nitrocellulose. For western blot the following antibodies were used: GRK2 (sc-13143), GAPDH (sc-365062), Gα i-1 (sc-13533) from SantaCruz Biotecnology; p53 (#2524S), Cytochrome c (# 4272S), LC3-II (# 2775S), Cleaved caspase 3 (# 9661S) from Cell signaling. In some experiments, cells were treated with 500 nM of 3-iodotyrosine (3-IY; Sigma #I8250), an inhibitor of TH enzyme, or with TH-siRNA (200 nM Ambion) to inhibit catecholamines synthesis in macrophages. SiRNA transfection was performed using lipofectamine 2000 (Invitrogen) following manufacturer instructions, and scramble-siRNA was used as control.

**Cell viability**

The assay was performed as described previously [15], using a CyQUANT® Assay Kit (Invitrogen #C35006) according to the manufacturer’s instructions. The H9C2 cells were plated in a 96-well plate (1 × 104 cells/well) and treated with D-Dox or M-Dox. After 24 hours the assay was performed to determine the number of viable cells. The data were acquired by using a plate reader (TecanInfinite200Pro), and the results are shown in the graph as the mean of three independent experiments.

**Mitochondrial transplantation**

Transplantation of isolated functional mitochondria was performed as previously described [16]. Briefly, mitochondria were isolated from untreated H9C2 by rupturing the cell pellet in homogenization buffer [HB; 20 mM HEPES-KOH (pH 7.4), 220 mM mannitol, and 70 mM sucrose] with 10 strokes of a 27-gauge needle. The homogenate was centrifuged at 400 × g; 5 min to remove unbroken cells, and the mitochondria pellet was then isolated by centrifugation at 5800 × g; 5 min. The number of isolated mitochondria was determined by using a Bio-Rad protein assay kit and expressed as protein concentration. The mitochondrial transfer was conducted by co-incubating isolated mitochondria (40 µg) with M-Dox treated H9C2 (1 × 105 cells/well of a 6-well plate) in 2 ml of the standard medium at 37°C under 5% CO2 for 24 hrs. A pilot experiment was conducted to determine the effectiveness of mitochondrial internalization, by staining the mitochondria of donor cells with mitotracker and evaluating the mitotracker signal in receiving cells.

**Proteomic analysis**

Trypsin digestion was performed as follows: cells were solubilized in 50 mM ammonium bicarbonate then vortexed gently and the proteins were reduced with 10 mM DTT for 30 min, then alkylated with 20 mM IAA for 1h in dark. The enzyme-substrate ratio was 1:100 the sample was incubated at 37°C overnight in a thermomixer comfort (Eppendorf); after centrifugation, the supernatant was then collected and used for MS analysis. A nanoflow ultra-high performance liquid chromatography (UHPLC) instrument (Ultimate 3000, Thermo Fisher Scientific, Bremen, Germany) was coupled online to an Orbitrap Lumos tribrid mass spectrometer (Thermo Fisher Scientific) with a nanoelectrospray ion source (Thermo Fisher Scientific). 1uL of the digest was first trapped on a PepMap trap column for 1.50 min at a flow rate of 30 μL/min (Thermo Fisher) and then peptides were loaded and separated onto a C18 reversed-phase column (25 cm x 75 μm I.D, 2.6m, BioZen Phenomenex, Bologna, Italy). Mobile phases were A: 0.1% HCOOH in water, B: 0.08%HCOOH in ACN/Water 80/20), and a linear 90 min gradient was performed. Each sample was analyzed by LC-MS/MS in triplicate. MS data were acquired using a data-dependent method, dynamically choosing the most abundant precursor ions from the survey scan (350–1500 m/z) using HCD fragmentation. Survey scans were acquired at a resolution of 120,000 at m/z 200. Unassigned precursor ion charge states, as well as singly charged species, were excluded. The isolation window was set to 3 Da a normalized collision energies NCE) of 27 was applied. Maximum ion injection times for MS and the MS/MS scans were 50 ms and 100 ms respectively, and ACG values were set to auto. Dynamic exclusion: 30 seconds. For data processing, raw MS data were analyzed using Proteome Discoverer software version 2.5 using the Sequest search engine and Inferys rescoring. The maximum allowed mass deviation was set to 10 ppm. Enzyme specificity was set to trypsin, and two missed cleavages were allowed. Carbamidomethylcysteine was set as a fixed modification, methionine oxidation, and phosphorylation of serine, threonine, and tyrosine as variable modifications. The spectra were searched against the Rattus Norvegicus sequence database from SWISSPROT database 11/2022, taxonomy id 10116) combined with 248 common contaminants and concatenated with the reversed versions of all sequences.

**Confocal microscopy analysis**

Mitochondrial morphology and membrane potential were assessed by high-resolution imaging using Nikon A1 confocal microscope equipped with a 60x 1.3NA oil immersion objective. For mitochondrial visualization, the cells were stained with mitotracker- green (150 nM), while tetramethylrhodamine (TMRE- 150 nM) was used as an indicator of mitochondrial potential [17]. At 24 hours after M-Dox treatment, the cells were incubated with a culture medium containing both dyes, at the indicated concentration, for 30 min at 37 c. Then, a live-cell imaging solution (HEPES buffered physiological saline, Invitrogen) was added and live cells were observed at a confocal microscope (Nikon A1). Fluorescence was excited with a 488 nm Argon laser (MitoTracker Green) and a 543 nm HeNe laser (TMRE). The analysis of co-localization of red (TMRE) and green (Mitotracker) signals was performed for each picture using Nikon software. Pearson’s coefficient was used as an indicator of the amount of healthy-polarized mitochondria (green and red-stained). The assessment of mitophagy was evaluated by adeno-associated virus (AAV) harboring Mito-Keima, a pH-sensitive reporter [18]. M-Dox treated cells were overnight infected with AAV-Mito-Keima (1:50). Live cells were observed at a confocal microscope (Nikon A1) and green vs red fluorescence was determined as an indicator of mitochondria delivered to lysosomes.

**Mitochondrial respiration**

Cells were treated with M-Dox and oxygen consumption was analyzed at Seahorse. The real-time oxygen consumption rate (OCR) of H9C2 was measured at 37 °C using a Seahorse XF Analyzer (Seahorse Bioscience, North Billerica, MA, USA). Cells were plated into specific cell culture microplates (Agilent, USA) at the concentration of 3 x 104 cells/well, and cultured for the last 24 h in a conditional medium derived from untreated or Dox-treated macrophages. OCR was measured in XF media (non-buffered DMEM medium, containing 10 mM glucose, 2 mM L-glutamine, and 1 mM sodium pyruvate) under basal conditions and after the sequential addition of 1.5 µM oligomycin, 2 µM FCCP, and rotenone+antimycin (0.5 µM all) (all purchased from Agilent).

**Statistical analysis**

Sample size evaluation was performed by G*Power software to achieve the statistical power of 0.8 based on our previous in vivo experiments [19]. All values are presented as mean ± SD. All experiments were performed at least in triplicate by blinded investigators. Unpaired t-test or ANOVA followed by Bonferroni post hoc testing were performed as appropriate, where applicable. A significance level of p < 0.05 was assumed for all statistical evaluations. Statistics were computed with GraphPad Prism Software (San Diego, California).

**References**

1. Tang XH, Gambardella J, Jankauskas S, Wang X, Santulli G, Gudas LJ, Levi R: **A Retinoic Acid Receptor beta 2 Agonist Improves Cardiac Function in a Heart Failure Model**. *J Pharmacol Exp Ther* 2021, **379**(2):182-190.

2. Ciccarelli M, Chuprun JK, Rengo G, Gao E, Wei Z, Peroutka RJ, Gold JI, Gumpert A, Chen M, Otis NJ *et al*: **G protein-coupled receptor kinase 2 activity impairs cardiac glucose uptake and promotes insulin resistance after myocardial ischemia**. *Circulation* 2011, **123**(18):1953-1962.

3. Ciccarelli M, Sorriento D, Franco A, Fusco A, Del Giudice C, Annunziata R, Cipolletta E, Monti MG, Dorn GW, 2nd, Trimarco B *et al*: **Endothelial G protein-coupled receptor kinase 2 regulates vascular homeostasis through the control of free radical oxygen species**. *Arterioscler Thromb Vasc Biol* 2013, **33**(10):2415-2424.

4. Hosseini NF, Amini R, Ramezani M, Saidijam M, Hashemi SM, Najafi R: **AS1411 aptamer-functionalized exosomes in the targeted delivery of doxorubicin in fighting colorectal cancer**. *Biomed Pharmacother* 2022, **155**:113690.

5. Jung BT, Jung K, Lim M, Li M, Santos R, Ozawa T, Xu T: **Design of 18 nm Doxorubicin-Loaded 3-Helix Micelles: Cellular Uptake and Cytotoxicity in Patient-Derived GBM6 Cells**. *ACS Biomater Sci Eng* 2021, **7**(1):196-206.

6. Siddiqui A, Gupta V, Liu YY, Nazzal S: **Doxorubicin and MBO-asGCS oligonucleotide loaded lipid nanoparticles overcome multidrug resistance in adriamycin resistant ovarian cancer cells (NCI/ADR-RES)**. *Int J Pharm* 2012, **431**(1-2):222-229.

7. Yousefpour P, Ahn L, Tewksbury J, Saha S, Costa SA, Bellucci JJ, Li X, Chilkoti A: **Conjugate of Doxorubicin to Albumin-Binding Peptide Outperforms Aldoxorubicin**. *Small* 2019, **15**(12):e1804452.

8. Zhang C, Zhang F, Han M, Wang X, Du J, Zhang H, Li W: **Co-delivery of 5-fluorodeoxyuridine and doxorubicin via gold nanoparticle equipped with affibody-DNA hybrid strands for targeted synergistic chemotherapy of HER2 overexpressing breast cancer**. *Sci Rep* 2020, **10**(1):22015.

9. Charlier B, Coglianese A, De Rosa F, Cozzolino A, Boccia G, Borrelli A, Capunzo M, Genovese G, De Caro F, Filippelli A *et al*: **A LC-MS/MS based methodology for the environmental monitoring of healthcare settings contaminated with antineoplastic agents**. *J Public Health Res* 2023, **12**(1):22799036231160629.

10. Liotti F, Kumar N, Prevete N, Marotta M, Sorriento D, Ierano C, Ronchi A, Marino FZ, Moretti S, Colella R *et al*: **PD-1 blockade delays tumor growth by inhibiting an intrinsic SHP2/Ras/MAPK signalling in thyroid cancer cells**. *J Exp Clin Cancer Res* 2021, **40**(1):22.

11. Sorriento D, Campanile A, Santulli G, Leggiero E, Pastore L, Trimarco B, Iaccarino G: **A new synthetic protein, TAT-RH, inhibits tumor growth through the regulation of NFkappaB activity**. *Mol Cancer* 2009, **8**:97.

12. Sorriento D, Fusco A, Ciccarelli M, Rungi A, Anastasio A, Carillo A, Dorn GW, 2nd, Trimarco B, Iaccarino G: **Mitochondrial G protein coupled receptor kinase 2 regulates proinflammatory responses in macrophages**. *FEBS Lett* 2013, **587**(21):3487-3494.

13. Iaccarino G, Izzo R, Trimarco V, Cipolletta E, Lanni F, Sorriento D, Iovino GL, Rozza F, De Luca N, Priante O *et al*: **Beta2-adrenergic receptor polymorphisms and treatment-induced regression of left ventricular hypertrophy in hypertension**. *Clin Pharmacol Ther* 2006, **80**(6):633-645.

14. Galasso G, De Rosa R, Piscione F, Iaccarino G, Vosa C, Sorriento D, Piccolo R, Rapacciuolo A, Walsh K, Chiariello M: **Myocardial expression of FOXO3a-Atrogin-1 pathway in human heart failure**. *Eur J Heart Fail* 2010, **12**(12):1290-1296.

15. Gambardella J, Fiordelisi A, Santulli G, Ciccarelli M, Cerasuolo FA, Sala M, Sommella E, Campiglia P, Illario M, Iaccarino G *et al*: **Exploiting GRK2 Inhibition as a Therapeutic Option in Experimental Cancer Treatment: Role of p53-Induced Mitochondrial Apoptosis**. *Cancers (Basel)* 2020, **12**(12).

16. Kitani T, Kami D, Matoba S, Gojo S: **Internalization of isolated functional mitochondria: involvement of macropinocytosis**. *J Cell Mol Med* 2014, **18**(8):1694-1703.

17. Song M, Mihara K, Chen Y, Scorrano L, Dorn GW, 2nd: **Mitochondrial fission and fusion factors reciprocally orchestrate mitophagic culling in mouse hearts and cultured fibroblasts**. *Cell Metab* 2015, **21**(2):273-286.

18. Shirakabe A, Fritzky L, Saito T, Zhai P, Miyamoto S, Gustafsson AB, Kitsis RN, Sadoshima J: **Evaluating mitochondrial autophagy in the mouse heart**. *J Mol Cell Cardiol* 2016, **92**:134-139.

19. Russo M, Guida F, Paparo L, Trinchese G, Aitoro R, Avagliano C, Fiordelisi A, Napolitano F, Mercurio V, Sala V *et al*: **The novel butyrate derivative phenylalanine-butyramide protects from doxorubicin-induced cardiotoxicity**. *Eur J Heart Fail* 2019, **21**(4):519-528.

**Supplementary Figure legends**

**Figure S1: Macrophage infiltration in the heart in response to Dox. A)** In vivo experimental model: Mice were treated with one intraperitoneal injection of Dox (20 mg/Kg). Echocardiographic analysis and ANP gene expression were performed at 0, 1, 3, and 5 days from Dox treatment. At 0 and 5 days hearts were collected to perform histological analysis. For flow cytometry, hearts were collected at 0, 1, 3, and 5 days. **B)** The expression levels of ANP were evaluated by Real-Time PCR in heart samples at the end of the treatment. The results are shown as mean±SEM in the bar graph; * p < 0.05 vs “time zero” (n=5). **C)** Mice were treated with intraperitoneal injections (3 injections/week) of Dox (2,5 mg/Kg). Paraffin-embedded sections of hearts were processed for immunofluorescence(magnification x400). Representative images of F4/80 staining reveal macrophage infiltration. **D)** To characterize macrophage phenotype, hearts were collected at five days from Dox treatment and the staining with specific markers of M1 and M2 (CD80, CD206, CCR2 and MHC-II) was evaluated by flow cytometry. Representative images are shown including a relative isotype control of CTRL group. The different percentages of macrophage phenotypes in response to Dox are shown in the graph based on CD80 and CD206 staining of CD45+ F4/80+ macrophages in control and Dox-treated mice are shown. A net polarization of macrophages towards M1 subtype (CD80^hi^, CD206^low^) compared with M2 (CD80^low^, CD206^hi^) is detected in Dox-treated hearts compared to control mice; * p < 0.05 vs M1-CTRL, # p < 0.05 vs M1-Dox (n=6).

**Figure S2: In vitro modelA)** Macrophages were treated with Dox for 24 hours and the cultured mediums from treated(M-Dox) and untreated(M-C) macrophages were transferred to H9C2 for additional 12/24 hours. In some experiments, H9C2 were directly treated with Dox(D-Dox) or were untreated as a control **B)** Macrophages were treated with Dox and cell viability was assessed at 12 and 24 hours to evaluate deleterious effects of Dox on these cells. Cell viability was reduced in a time-dependent manner; * p < 0.05 vs Basal, # p < 0.05 vs 12 hrs. **C)** Dox was removed from the conditioned culture medium before treating H9C2. The absence of Dox in M-Dox was evaluated by mass spectrometry in which we analyzed the fresh medium plus doxorubicin, as control (1), the non-filtered M-Dox medium which includes free Dox (2), and the filtered M-Dox medium (3). In filtered M-Dox medium no Dox signal was detected, suggesting that free Dox in the medium was removed.

**Figure S3: p53 interactome.** It is reported a STRING interaction graph of p53 interacting proteins was identified by mass spectrometry analysis. Network nodes represent proteins with their UNIPROT ID accession: Rpl7a: Ribosomal protein L7a; Hspd1: 60 kDa heat shock protein; Tp53: Cellular tumor antigen p53, Casp3: Caspase-3; Hist1h2bh: Histone H2B; Hspb1: Heat shock protein family b; Ywhaz: 14-3-3 protein zeta/delta; Ywhaq: theta; 14-3-3 protein theta; Rpl29: 60S ribosomal protein L29; Ppia: Peptidyl-prolyl cis-trans isomerase a; Hist1h1d: Histone H1.4; Gapdh: Glyceraldehyde-3-phosphate dehydrogenase; Map1lc3b: Microtubule-associated proteins 1A/1B light chain 3B; LOC679794: Cytochrome c, somatic-like; Rps6: 40S ribosomal protein S6; Eno1: Alpha-enolase; Actb: Actin, cytoplasmic 1; Map1lc3a Microtubule-associated proteins 1A/1B light chain 3A; Casp12: Caspase 12; Vim: Vimentin; LOC688948: Ribosomal protein S26; Eef2: Elongation factor 2; ENSRNOP00000025380: Small subunit ribosomal protein s28e. Edges represent protein-protein interactions and edge lines color and thickness represent confidence (large dark gray: highest= 0.900, thick light gray: lowest= 0.150).

**Figure S4: Alterations of mitophagy in M-Dox treated cells. A)** Cells were treated with Urolithin-A and LC3-II was evaluated by western blot. The treatment with Urolithin-A induced autophagic flux. The bar graph shows a densitometric analysis; * p<0.05 vs M-Dox. **B)** Cells were treated with Urolithin-A and cleaved caspase 3 levels were evaluated by western blot. The treatment with Urolithin-A reduced caspase 3 levels in response to M-Dox. The bar graph shows a densitometric analysis; * p<0.05 vs M-C. **C)** Mitochondria from healthy H9C2 were transplanted in M-Dox treated cells. The image shows mitotracker staining of mitochondria. Apoptosis was evaluated by western blot and densitometric analysis is shown in the bar graph; * p<0.05 vs M-C. The results are the mean of three independent experiments.

**Figure S5: Mitochondrial morphology in D-Dox treated cells.** H9C2 were treated with D-Dox or M-Dox and incubated with Mitotracker (Invitrogen) accordingly to manufacturer instructions. The mitochondrial structure was visualized at Nikon Eclipse fluorescent microscopy.

**Figure S6: TH gene deletion in macrophages.** Specific TH siRNA or scramble siRNA as control were transfected in RAW264.7. TH expression was evaluated by western blot. The mouse adrenal gland was used as a positive control. The bar graph shows a densitometric analysis; * p<0.05 vs CTRL.

**Figure S7: Effects of adrenergic modulation in cardiomyoblasts**. **A**) H9C2 were treated with different doses of epinephrine and mitochondrial localization of p53 was analyzed by western blot. The bar graph shows a densitometric analysis; * p<0.05 vs “0 ng”. **B**) H9C2 were treated with metoprolol (10µM) and caspase 3 levels were analyzed by western blot. The bar graph shows a densitometric analysis; * p<0.05 vs M-C, ! p<0.05 vs M-Dox. **C-D)** GRK2 levels were evaluated by western blot in the membrane (C) and mitochondrial (D) extracts from cells treated with M-Dox. The kinase levels were increased in response to M-Dox in the plasma membrane and were reduced in mitochondria. The bar graph shows a densitometric analysis; * p<0.05 vs M-C.

**Figure S8: Effects of M-Dox in heart samples and cardiomyocytes. A-B)** Total levels of GRK2 were evaluated by western blot in hearts samples from mice at the end of the treatment (A) and in cardiomyocytes isolated from mice hearts and treated with M-Dox (B). GRK2 levels decreased in both heart samples and cardiomyocytes. Data are shown as Mean±SEM in the bar graph; * p<0.05 vs CTRL (A) and M-C (B). **C-D)** Mitochondrial levels of p53 and cytosolic release of cytochrome c were evaluated by western blot in hearts samples from mice at the end of the treatment (C) and in cardiomyocytes isolated from mice hearts and treated with M-Dox (D). Mitochondrial p53 and cytosolic cytochrome c levels were increased in both heart samples and cardiomyocytes. Data are shown as Mean±SEM in the bar graph; * p<0.05 vs CTRL(C) and M-C (D). **E-F**) Mitochondrial biogenesis was evaluated by Real-Time PCR in hearts samples from mice at the end of the treatment (E) and in cardiomyocytes isolated from mice hearts and treated with M-Dox (F). Mitochondrial biogenesis was reduced in hearts treated with Dox in a time-dependent manner (E). It was increased in cardiomyblasts in response to D-Dox, but it remains unchanged (blocked) in response to M-Dox. Data are shown as Mean±SEM in the bar graph; * p<0.05 vs “0” and D-C.

**Figure S9: Effects of Dox in peritoneal macrophages. A)** Peritoneal macrophages were characterized by flow cytometry. Representative images of the staining for CD45, F4/80, CD80, CD206, CCR2 and MHC-II are shown.- **B**) Both norepinephrine and epinephrine production wa smeasured by ELISA assay in the cultured medium of peritoneal macrophages treated with Dox. Catecholamine levels were increased as it occurs in RAW264.7; * p<0.05 vs M-C. **C-D)** Mitochondrial levels of p53 and cytosolic release of cytochrome c were evaluated by western blot in H9C2 in response to conditioned medium from peritoneal macrophages treated with Dox (24 hours). Mitochondrial p53 (C) and cytosolic cytochrome c (D) levels increased, confirming data with RAW264.7. Data are shown as Mean±SEM in the bar graph; * p<0.05 vs and M-C.

**Central Illustration: Dox-activated macrophages exacerbates cardiac damage in response to Dox.** Dox induces early recruitment of macrophages in the heart which, in turn, release catecholamines and activate cardiac β-ARs. This induces p53-dependent damage in mitochondria that culminate with the activation of the caspases cascade and cell death.

**
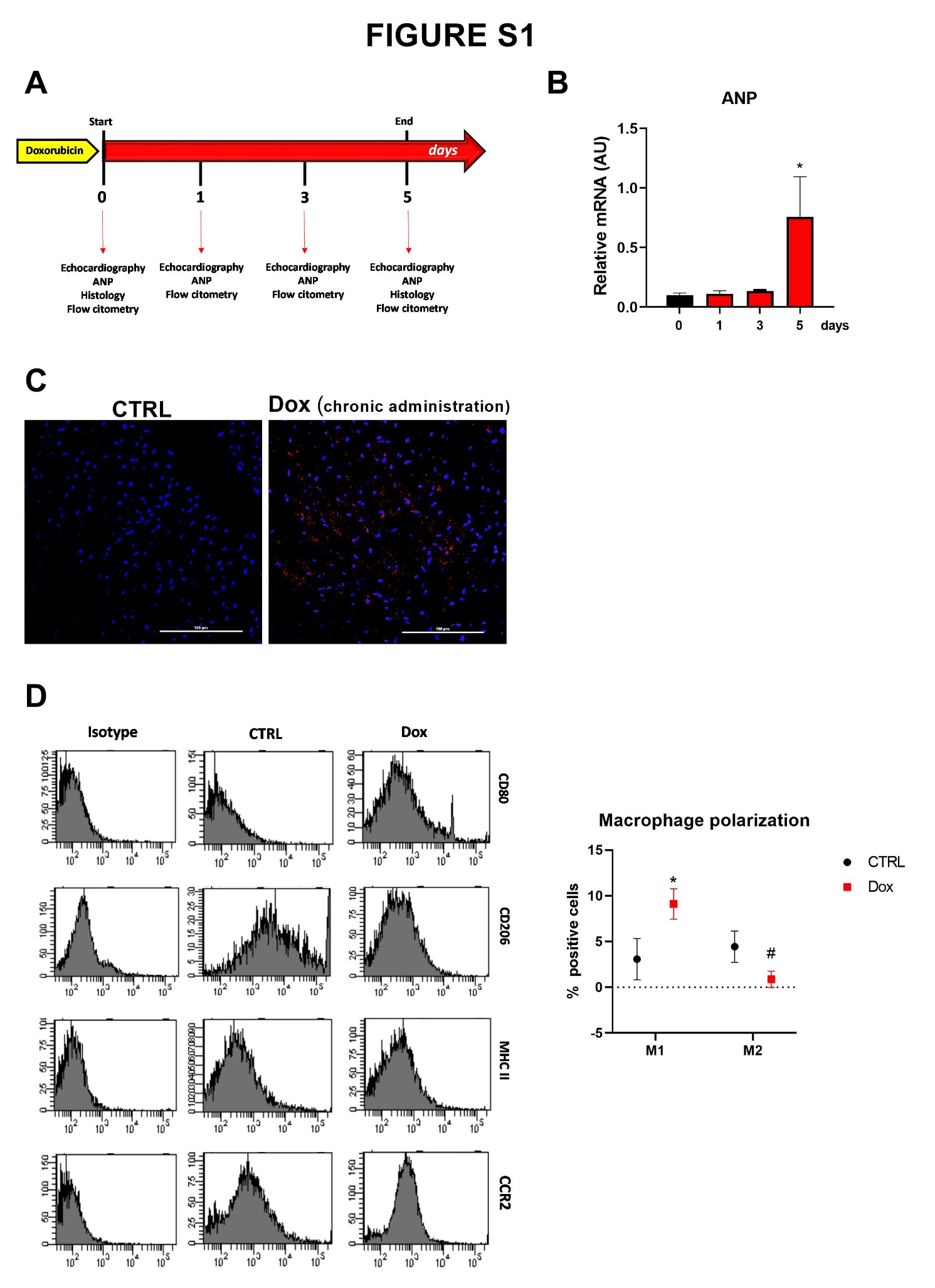

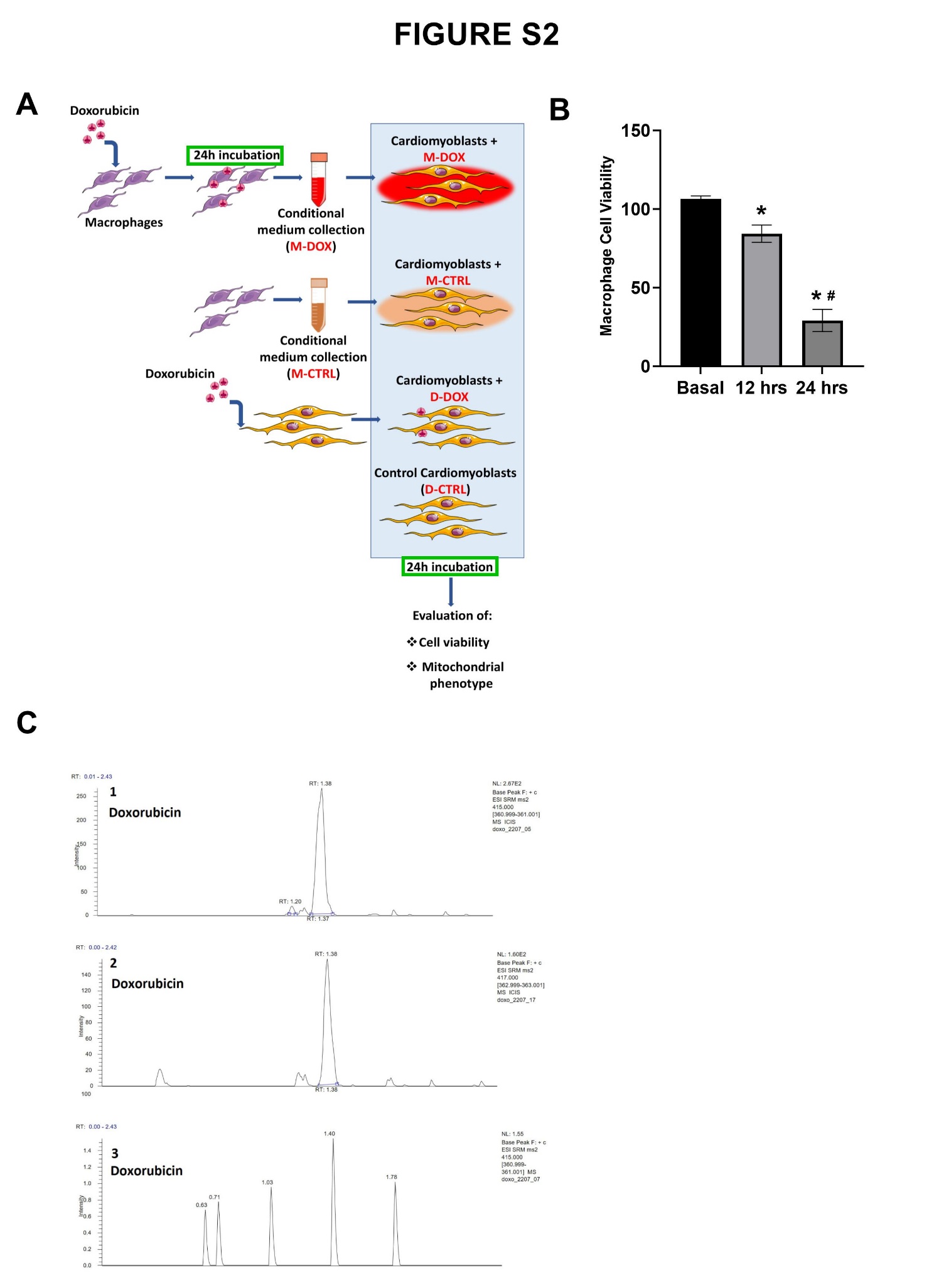

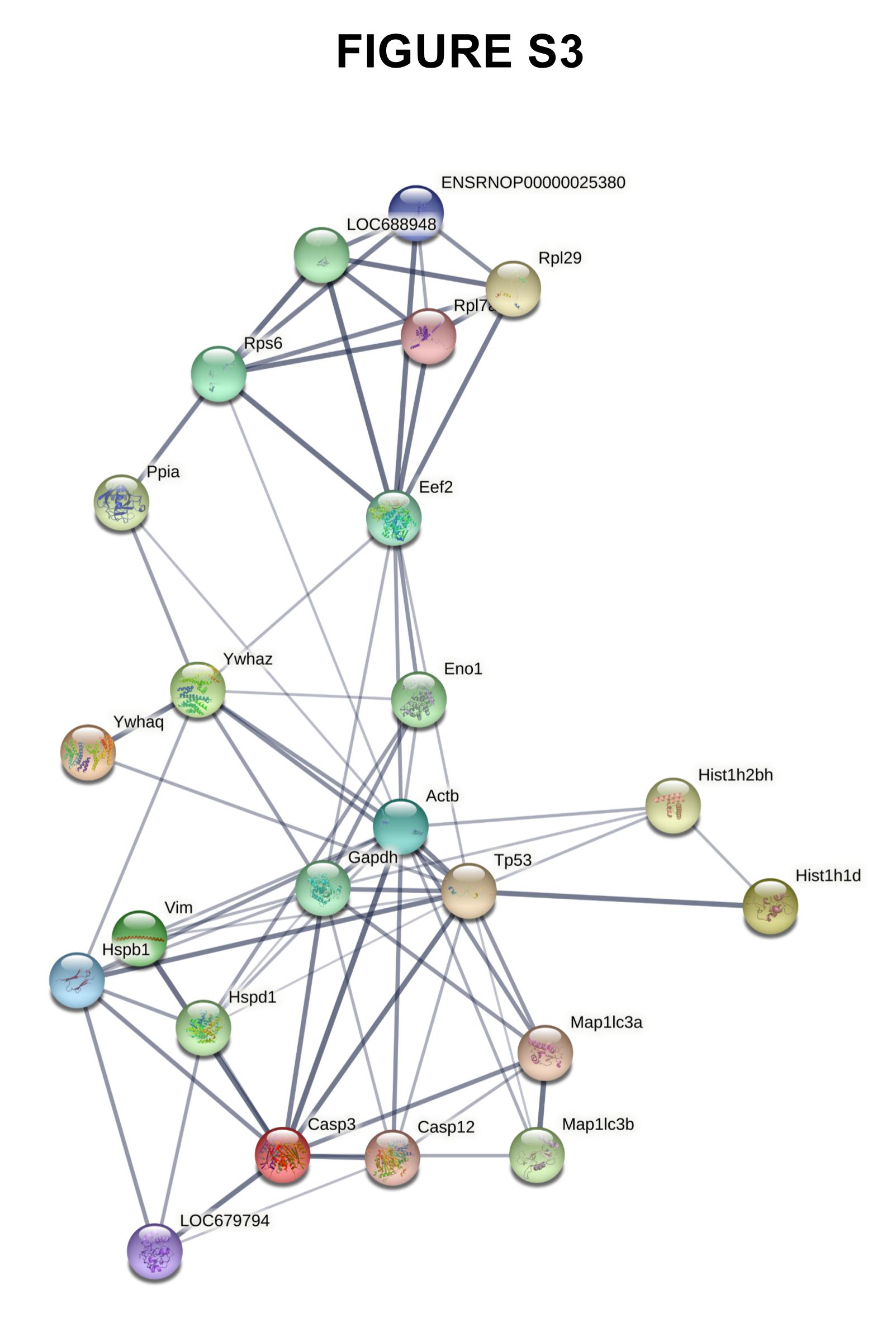

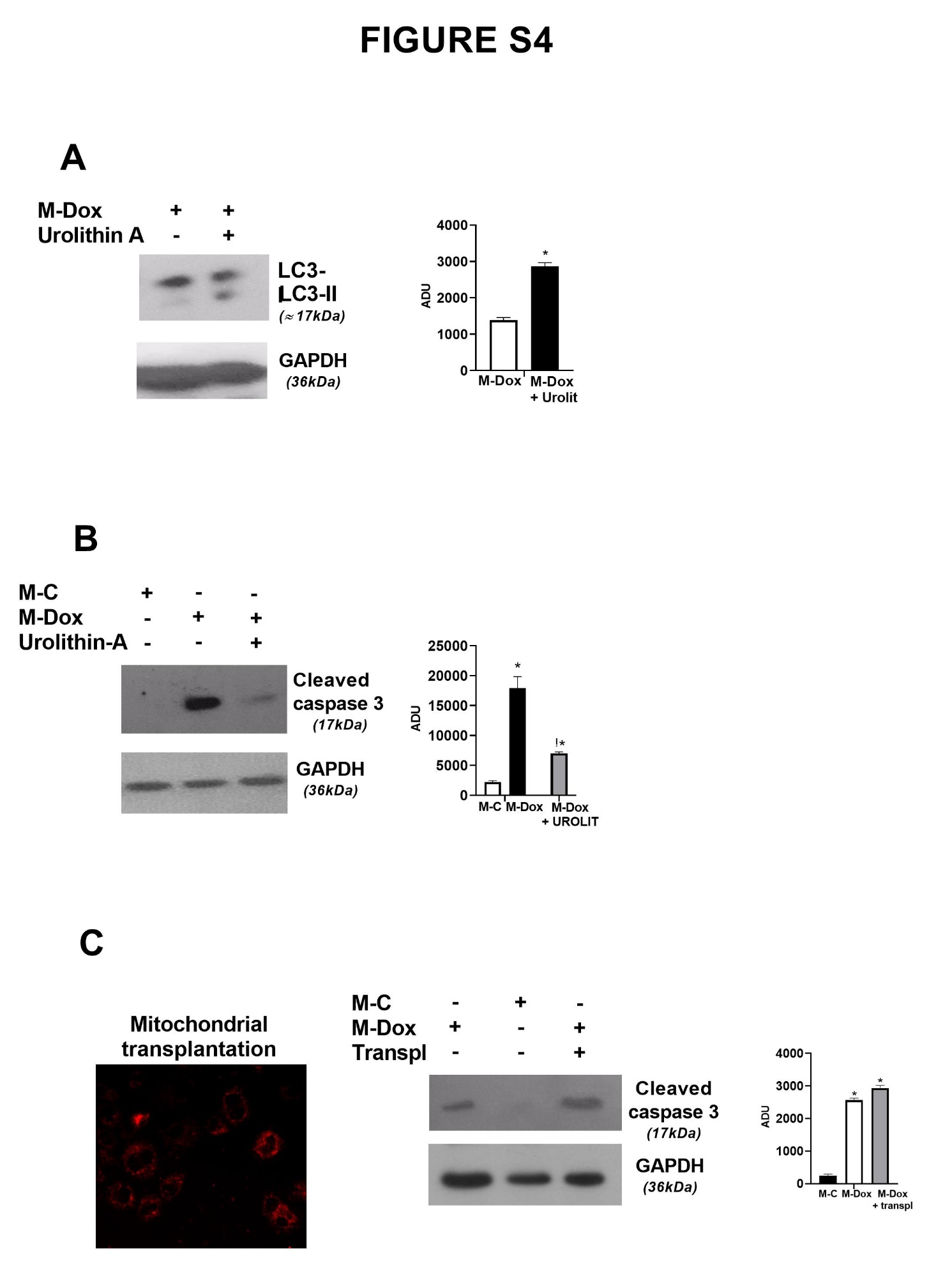

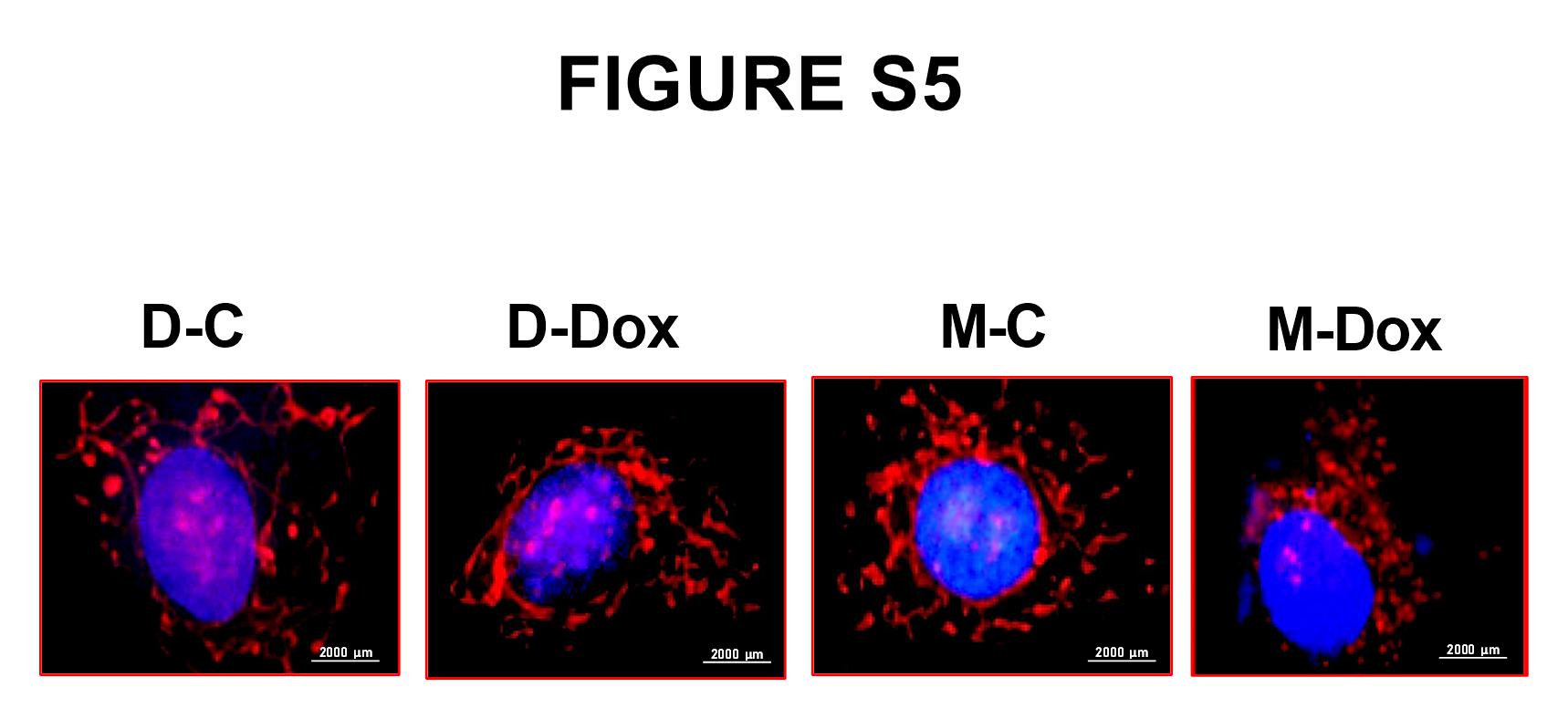
**

**
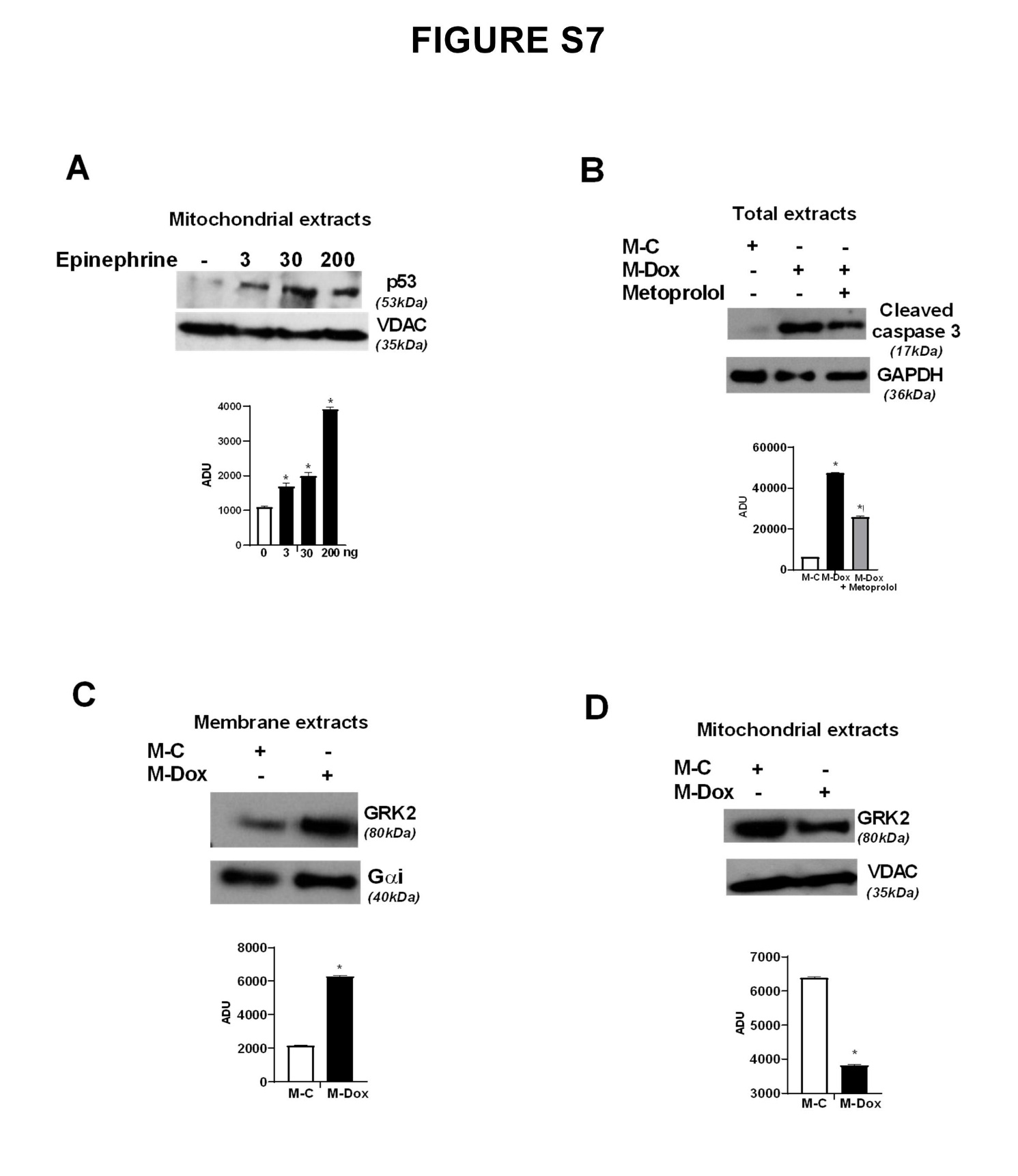
**

**
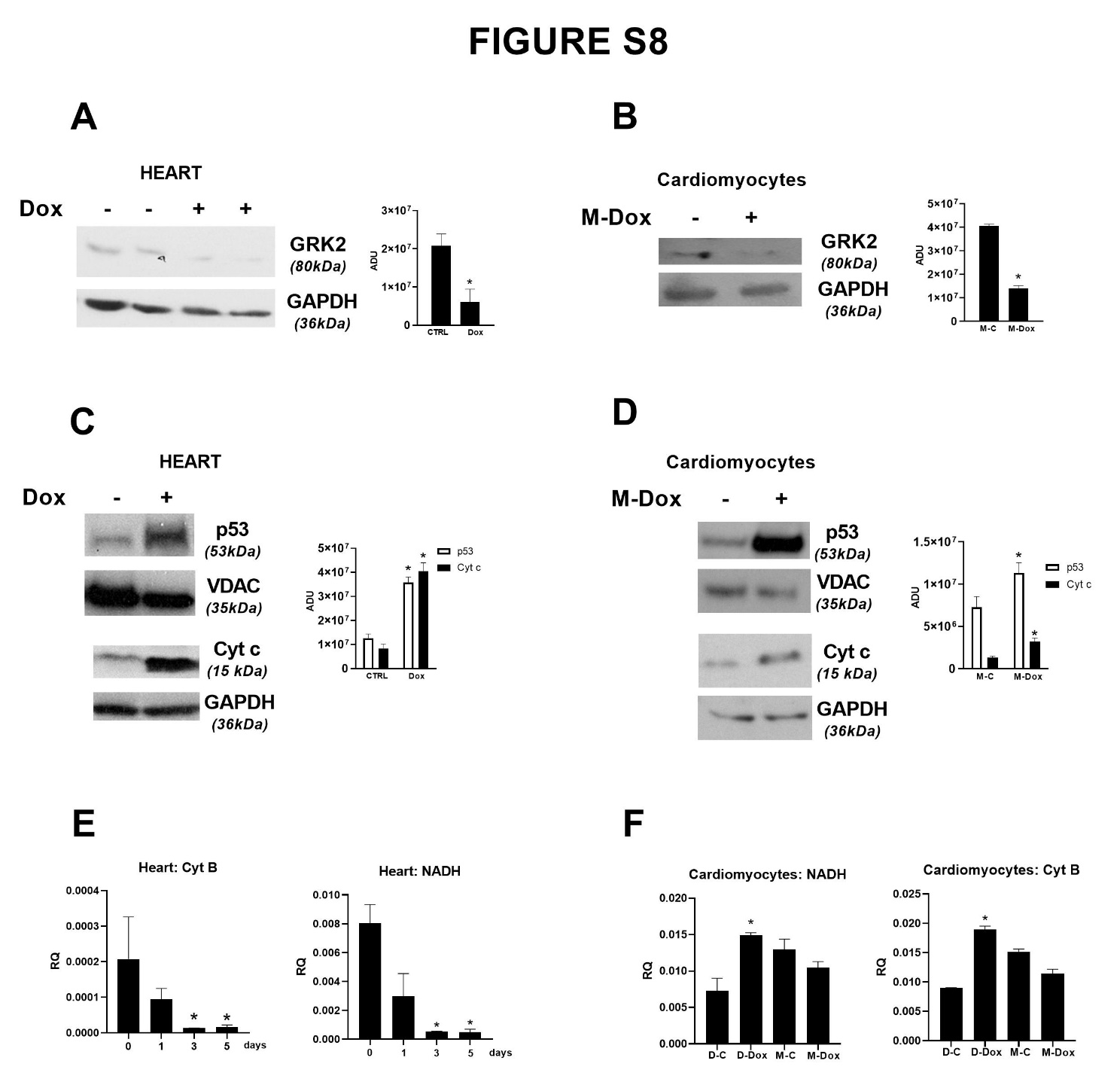
**

**
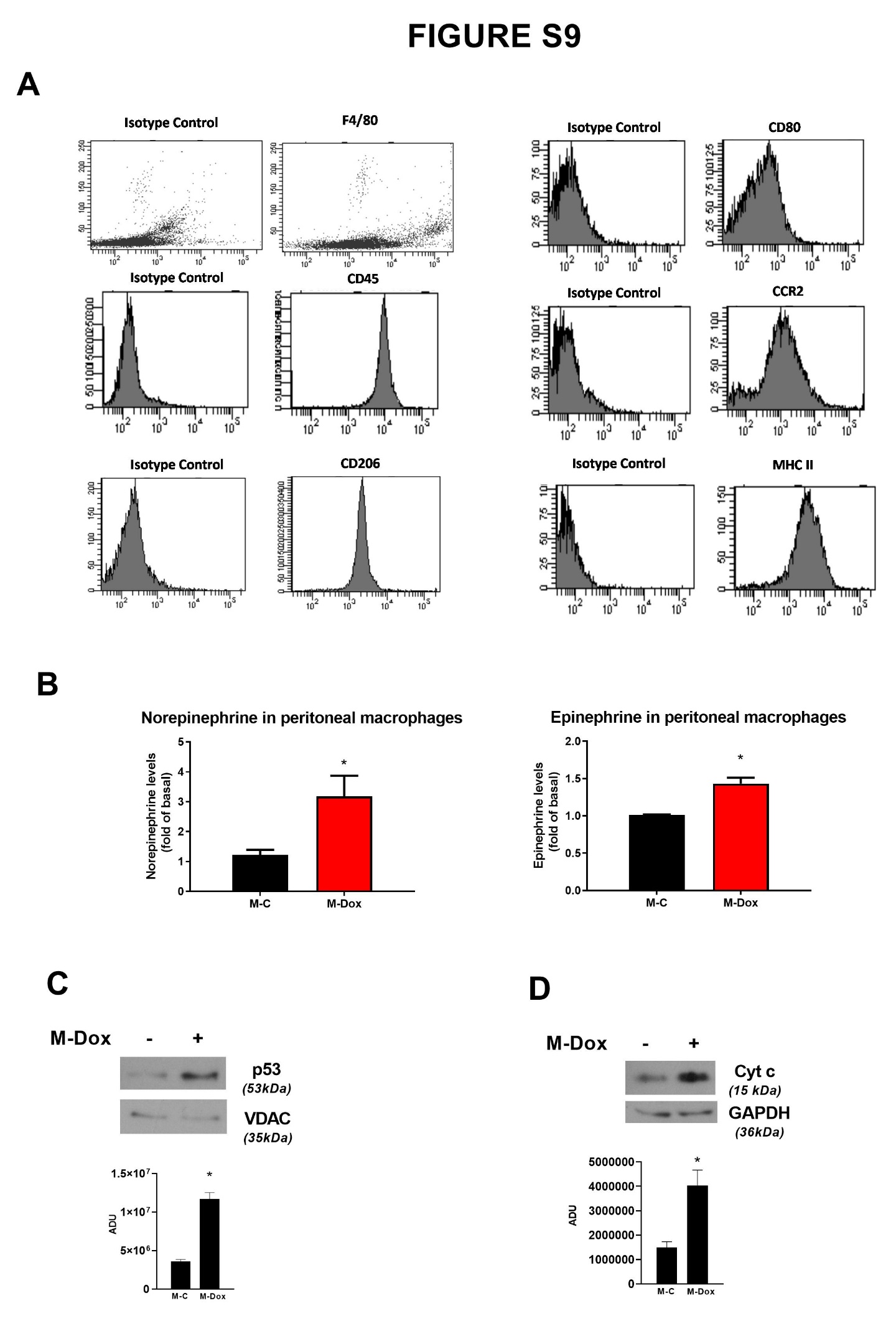
**

**Supplementary Table 1**

**Average normalized intensity plus CV% of proteins involved in metabolic processes exhibiting differential expression in cell lysates from M-Dox and M-C groups.**

| Accession | Description | Avg. Normalized Abundance CTRL | Avg. Normalized Abundance M-Dox |
| --- | --- | --- | --- |
| P68255 | 14-3-3 protein theta | 2.39E^+07^±16.69 | 3.89E^+07^±20.32 |
| P63102 | 14-3-3 protein zeta/delta | 6.95E^+07^±8.53 | 9.14E^+07^±8.56 |
| P60711 | Actin, cytoplasmic 1 | 1.39E^+09^±14.94 | 1.93E^+09^±2.87 |
| P15865 | Histone H1.4 | 6.94E^+08^±10.27 | 3.82E^+08^±7.64 |
| P42930 | Heat shock protein beta-1 | 1.02E^+08^±16.80 | 1.06E^+08^±17.77 |
| P05197 | Elongation factor 2 | 4.49E^+08^±5.03 | 5.77E^+08^±4.30 |
| P04797 | Glyceraldehyde-3-phosphate dehydrogenase | 4.11E^+08^±10.14 | 4.45E^+08^±7.54 |
| P31000 | Vimentin | 2.05E^+09^±3.52 | 1.92E^+09^±2.44 |
| P04764 | Alpha-enolase | 1.72E^+08^±11.11 | 1.99E^+08^±3.69 |
| P62859 | 40S ribosomal protein S28 | 3.90E^+07^±18.91 | 2.81E^+07^±20.14 |
| P25886 | 60S ribosomal protein L29 | 2.38E^+07^±16.98 | 1.36E^+07^±5.62 |
| P62425 | 60S ribosomal protein L7a | 1.44E^+08^±5.46 | 8.08E^+07^±3.66 |
| P10111 | Peptidyl-prolyl cis-trans isomerase A | 2.34E^+08^±8.01 | 3.06E^+08^±7.28 |
| P62856 | 40S ribosomal protein S26 | 4.55E^+07^±12.85 | 3.15E^+07^±6.98 |
| P62755 | 40S ribosomal protein S6 Accession | 9.19E^+07^±4.11 | 5.84E^+07^±6.67 |
| Q00715 | Histone H2B type 1 | 1.52E^+09^±18.73 | 7.65E^+08^±20.32 |
| P63039 | 60 kDa heat shock protein, mitochondrial | 1.94E^+08^±11.71 | 1.75E^+08^±4.26 |
| P62898 | Cytochrome c | 2.78E^+07^±14.19 | 3.09E^+07^±20.43 |
| P55213 | Caspase-3 | 1.37E^+06^±18.37 | 3.53E^+05^±20.74 |
| Q920D5 | Caspase-12 | 7.47E^+05^±1.00 | 4.52E^+05^±8.90 |
| Q6XVN8 | Microtubule-associated proteins 1A/1B light chain 3A | 9.31E^+05^±19.47 | 1.50E^+06^±15.87 |
| Q62625 | Microtubule-associated proteins 1A/1B light chain 3B | 9.36E^+05^±18.47 | 7.92E^+05^±20.86 |
